# Physical stimuli-responsive polymeric patches for healthcare

**DOI:** 10.1016/j.bioactmat.2024.08.025

**Published:** 2024-09-28

**Authors:** Yifan Cheng, Yuan Lu

**Affiliations:** a Department of Chemical Engineering, Tsinghua University, Beijing, 100084, China; b Key Laboratory of Industrial Biocatalysis, Ministry of Education, Tsinghua University, Beijing, 100084, China

**Keywords:** Polymer patches, Physical stimulus, Healthcare, Therapy, Intelligence

## Abstract

Many chronic diseases have become severe public health problems with the development of society. A safe and efficient healthcare method is to utilize physical stimulus-responsive polymer patches, which may respond to physical stimuli, including light, electric current, temperature, magnetic field, mechanical force, and ultrasound. Under certain physical stimuli, these patches have been widely used in therapy for diabetes, cancer, wounds, hair loss, obesity, and heart diseases since they could realize controllable treatment and reduce the risks of side effects. This review sketches the design principles of polymer patches, including composition, properties, and performances. Besides, control methods of using different kinds of physical stimuli were introduced. Then, the fabrication methods and characterization of patches were explored. Furthermore, recent applications of these patches in the biomedical field were demonstrated. Finally, we discussed the challenges and prospects for its clinical translation. We anticipate that physical stimulus-responsive polymer patches will open up new avenues for healthcare by acting as a platform with multiple functions.

## Highlights

• The basic design principles of polymer patches are summarized.
• The most used physical stimuli for controllable treatment are summarized.
• The fabrication and characterization methods of patches are introduced.
• The biomedical applications of polymer patches are systematically overviewed.
• The challenges and prospects of polymer patches are discussed.

## Graphical abstract

**Figure:**
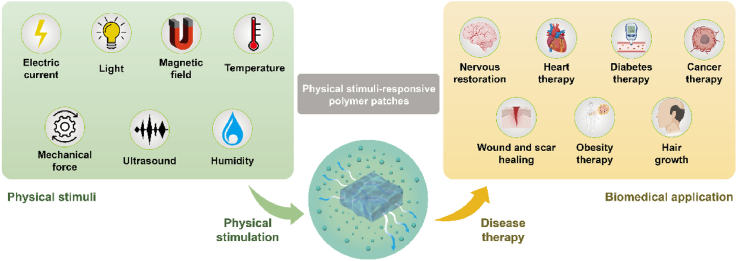

**Table:** Abbreviation Index

Abbreviation	Full name
SEBS	styrene-block-(ethylene-co-butylene)-block-styrene
PCL	Polycaprolactone
PVP	polyvinylpyrrolidone
HPMC	hydroxypropyl methylcellulose
ALG	Alginate
CTS	Chitosan
HA	hyaluronic acid
MN	Microneedle
ROS	reactive oxygen species
NIR	near-infrared
UV	Ultraviolet
DOX	Doxorubicin
ICG	indocyanine green
PTT	photothermal therapy
DDS	drug delivery system
DDPTA	debonding-on-demand polymeric tissue adhesive
ICE	ionic conducting elastomer
PPy	poly-pyrrole
Dex	dexamethasone
PENG	piezoelectric nanogenerator
TENG	triboelectric nanogenerator
ET	electrotransfection
ES	electrostimulation
CuO_2_	copper peroxide
OV-TiO_2_	oxygen vacancy-rich porous titanium oxide
PDMS	polydimethylsiloxane
PVA	polyvinyl alcohol
RJS	rotary jet spinning
PCM	phase-change material
EGF	epidermal growth factor
CPH	conducting polymer hydrogel
PEG	polyethylene glycol
CNT	carbon nanotubes
PLA	poly (lactic acid)
MI	myocardial infarction
VEGF	vascular endothelial growth factor
PHCuS NPs	porous hollow copper sulfide nanoparticles
AuNRs	gold nanorods
MACP	magnetically active cardiac patch
MAS	magnetic actuation system
GLP-1	glucagon-like peptide-1
PDA	polydopamine
LA	lauric acid
SA-g-NIPAM	sodium alginate-g-N-isopropyl acrylamide
DS	diclofenac sodium
PDT	photodynamic therapy
TA-Fe	tannic acid-Fe
BBH	berberine hydrochloride
SNP	sodium nitroprusside
MTX	methotrexate
LMWH	low molecular weight heparin
SMNs	silk fibroin MNs
PB	Prussian blue
CDDP	Cisplatin
CINP	cuttlefish ink
MHT	magnetic hyperthermia therapy
WAT	white adipose tissue
BAT	brown adipose tissue
Rosi	Rosiglitazone
CuS-NDs	copper sulfide nanodots
Mx	Minoxidil
ADSC-NVs	adipose-derived stem cell nanovesicles
JAM-A	junctional adhesion molecule A
rhGH	recombinant human growth hormone
MPs	microspheres
BP	black-phosphorus
GT	Gelatin
AI	artificial intelligence
4D	4-dimensional
P (AAm-co-VI)	poly (acrylamide-co-1-vinyl imidazole)
QCS	quaternary ammonium salt chitosan
HS	hypertrophic scarring
HSF	hypertrophic scar fibroblast
ALA	aminolevulinic acid
ALA-ES	ALA-loaded nanoethosome
DC	direct current
EF	electric field

## 1 Introduction

Long-term healthcare, particularly the treatment of chronic diseases, has received increasing attention in recent years. With the rapid advancement of global socioeconomics, the incidence of chronic conditions such as diabetes [[1], [2], [3]], infected wounds [[4], [5], [6]], cancer [[7], [8], [9]], dermatological diseases [[10], [11], [12]], and hair loss [[13], [14], [15]] has risen significantly. These conditions have emerged as some of the most serious public health challenges of the 21st century, negatively impacting the daily lives of many patients. As a result, developing effective and safe treatment methods has become an urgent priority. Unlike emerging infectious diseases, chronic conditions typically require long-term treatment, which must be tailored to the individual needs of each patient [16,17]. Traditional therapeutic strategies, such as drug injections, carry risks, including epidermal damage, permanent nerve injury, and poor treatment adherence [18,19]. Consequently, there is a growing need for innovative treatment methods in healthcare. One promising approach involves the development of biomedical patches using functional polymers with high biocompatibility. These polymer patches offer several advantages, including biocompatibility, processability, and cost-effectiveness, making them a highly promising option for healthcare applications [[20], [21], [22]].

Various polymer materials are currently being utilized to design these patches, such as cellulose [23], dextran [24], chitosan [25], acrylic polymers [26], SEBS copolymers [27], PCL [28], PVP [29], sodium alginate [30], and pullulan [31]. These patches have been used to deliver drugs for the treatment of diabetes [32], cancer [33], wounds [34], and hair loss [35]. However, drug delivery through these patches typically occurs via direct drug release or the deconstruction or degradation of drug-carrying particles. This method may not meet the requirements for on-demand treatment without external regulation, potentially leading to suboptimal therapeutic outcomes or the risk of drug overdose [36]. To address these issues, physical stimulus-responsive patches have been developed to enable precise, on-demand treatment, thereby reducing the adverse effects of off-target therapies.

**Fig. 1:**
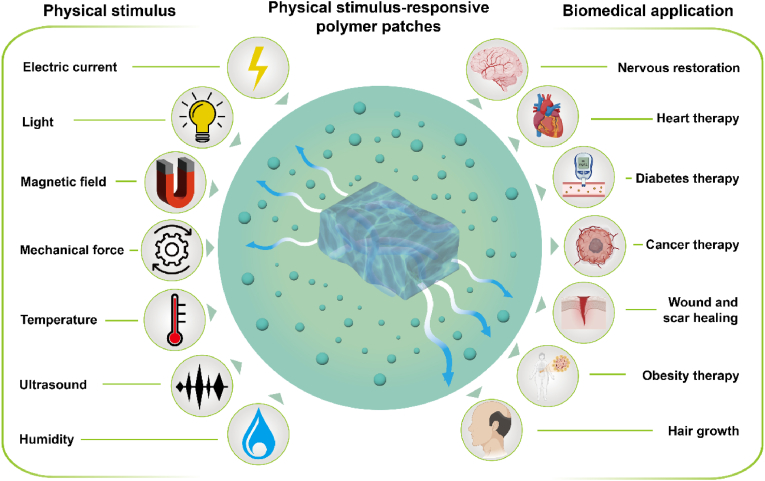
Schematic of the physical stimulus used in polymer patches and the biomedical application of physical stimulus-responsive polymer patches. Physical stimulus, including light, electric current, temperature, magnetic field, mechanical force, and ultrasound, can be utilized to control the treatment process. Such polymer patches can be used for nervous system restoration, heart therapy, diabetes therapy, cancer therapy, wound healing, obesity therapy, and hair growth.

Physical stimulus-responsive polymer patches can respond to various external stimuli, such as light [[37], [38], [39]], electric currents [[40], [41], [42]], temperature changes [[43], [44], [45]], magnetic fields [[46], [47], [48]], mechanical forces [[49], [50], [51]], ultrasound [52] and humidity [53] (Fig. 1). By applying these physical stimuli, the treatment process can be precisely controlled, reducing side effects. Light-responsive patches often contain photosensitive or thermosensitive agents that convert light into heat to regulate the treatment process. Electric current-responsive patches can undergo reversible redox reactions and structural changes under an electric field, allowing for controlled drug delivery. Thermo-responsive patches can undergo phase changes at specific temperatures, altering the polymer structure and controlling drug release. Magnetic field-responsive patches typically contain metal-based nanoparticles that can be guided by a magnetic field or convert the field's energy into heat to control the treatment process. Mechanical force-responsive patches can respond to mechanical strains in muscles, tendons, bone joints, and other organs, triggering structural changes under specific forces. Ultrasound-responsive patches can utilize the acoustic energy generated by ultrasound stimulation to disrupt the self-assembly balance of polymers, leading to polymer dissolution and drug release. Humidity-responsive patches could undergo volumetric change under humidity conditions, which can be used to control the process of treatment. All these physical stimuli can be employed for self-regulated healthcare treatments.

Numerous studies have focused on using physical stimulus-responsive polymer patches for long-term healthcare, particularly in disease monitoring and controlled drug delivery. In addition to drug delivery, these patches have also garnered attention for their potential in physical therapy applications, such as phototherapy and hyperthermia. Some studies have explored combining different therapeutic approaches to enhance treatment efficacy and minimize side effects. Furthermore, combining different physical control methods, such as light and electric fields, has been widely investigated to improve the precision of treatment. Additionally, efforts have been made to develop polymer patches with additional functionalities, such as monitoring, self-powering, and energy storage, which could enable more straightforward and precise treatments for patients. These advances are making polymer patches increasingly “smart,” with more sophisticated capabilities.

While many reviews have focused on the design and application of functional polymer materials for healthcare, comprehensive overviews specifically addressing physical stimuli-responsive polymer patches remain scarce. This review aims to provide an in-depth examination of recent developments in physical stimuli-responsive polymer patches for healthcare. These patches respond to external physical stimuli, including light, electric currents, magnetic fields, temperature changes, mechanical forces, and ultrasound, and these stimuli can be utilized for precise, on-demand treatment of conditions such as diabetes, cancer, wounds, hair loss, obesity, and heart disease. The review also addresses current challenges associated with using polymeric patches as drug depots in the biomedical field and concludes with a summary of the challenges, opportunities, and future perspectives for physical stimuli-responsive polymer patches in biomedicine.

## 2 Polymer patch design

**Fig. 2:**
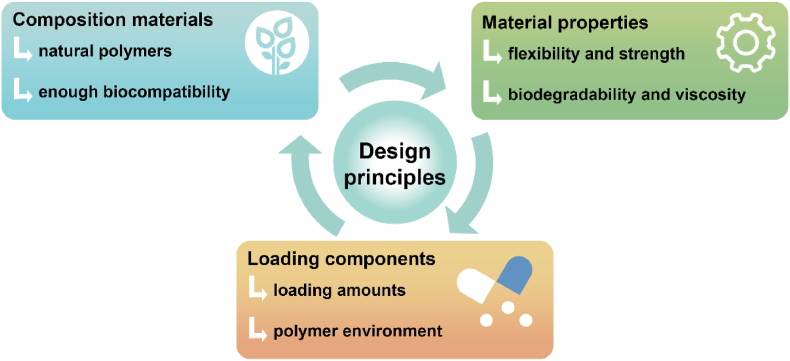
The design principles of physical stimulus-responsive polymer patches, including composition materials, material properties, and loading components.

The characteristics and functions of polymer patches are determined by their composition, material properties, and loading components. The composition directly affects the biocompatibility of the patches, which is crucial for ensuring treatment safety. Material properties, such as flexibility in non-invasive patches and viscosity in invasive patches, influence their practical application. The loading components are critical to the therapeutic effectiveness of the patches, and the impact of the polymer environment on these components must also be considered. Therefore, the development of polymer patches with specific healthcare functions requires careful consideration of composition, material properties, and loading components. This section summarizes the design principles essential for creating functional polymer patches (Fig. 2).

### 2.1 Composition

Since these patches are intended for healthcare applications, biocompatibility is a primary concern for user safety. This section introduces polymers with excellent biocompatibility, which are commonly used in the design of functional polymer patches. Most of these materials are derived from natural polymers to ensure adequate biocompatibility and treatment safety.

Cellulose, the most abundant biopolymer found in nature, offers several advantages, including low cost, high abundance, biocompatibility, biodegradability, and nontoxicity [54]. However, cellulose's insolubility in water and conventional solvents poses a significant challenge [55]. To address this, cellulose is often functionalized through chemical modifications of its hydroxyl groups [56,57]. For instance, methylation of hydroxyl groups produces HPMC, which can be used as a drug delivery matrix [58,59].

ALG, a polysaccharide derived from brown algae, is another widely used polymer in the biomedical field [60]. ALG is valued for its biocompatibility, porosity, water retention capacity, and tunable viscosity [60,61]. Moreover, due to ion exchange between monovalent ions in the alginate solution and polyvalent cations, ALG can serve as a conductive polymer in certain functional patches [62]. However, this ion exchange can lead to uncontrolled polymer dissolution [63], so ALG is often combined with other polymers to enhance its mechanical properties [64].

CTS, the second most abundant natural polymer after cellulose, is a biocompatible, bifunctional, and biodegradable amino polysaccharide. CTS exhibits excellent biological properties, making it suitable for applications in biomedicine, pharmaceuticals, tissue engineering, drug delivery, and membrane production [65]. However, its poor mechanical characteristics, low water resistance, and limited thermal stability [66] mean that CTS is typically blended with other synthetic or natural polymers [67].

HA, a glycosaminoglycan found in the extracellular matrix (ECM) of most tissues, particularly cartilage and ocular tissues [68], is renowned for its biodegradability, bioactivity, and biocompatibility. However, HA's rapid degradation and poor mechanical properties limit its biomedical applications. To overcome these challenges, various strategies, such as functionalization and cross-linking, have been developed to improve its stability and physicochemical properties [69].

Gelatin, a natural protein-derived material, is widely used in the biomedical field due to its biodegradability, non-immunogenicity, and commercial availability [70]. The presence of an arginine-glycine-asparagine sequence in gelatin promotes cell adhesion and migration, making it an ideal material for tissue repair [71]. However, gelatin-based patches suffer from limitations such as poor adhesion and susceptibility to tearing. To address these issues, functional groups like catechol are often introduced into the polymer chain to enhance adhesion. The self-healing ability of gelatin relies on specific reactions, such as the formation of reversible chemical bonds.

Other polymers commonly used in biomedical patches, such as polyvinylpyrrolidone [29], polycaprolactone [72], and acrylic polymers [26], also offer various advantages. In summary, a wide range of biocompatible polymers has been extensively utilized in biomedical patches. These polymers not only possess inherent benefits but can also be combined to enhance specific characteristics for different applications.

### 2.2 Properties

Polymer patches can be classified into two main categories based on their application: non-invasive and invasive patches. Non-invasive patches adhere to external tissues such as the skin [73], gastric mucosa [74], or nasal mucosa [75], while invasive patches are designed to adhere to internal organs, including the heart [76], brain [77], and liver [78]. Due to their distinct applications, these two types of patches exhibit different properties.

For non-invasive patches, flexibility is crucial to accommodate the elasticity of skin and other tissues [79]. Patches made from soft materials can minimize discomfort during use. Additionally, the adhesiveness of these patches must be carefully considered; patches with excessive adhesiveness may cause pain and trauma during removal [80], making them unsuitable for fragile or sensitive skin, such as that of infants or diabetic patients [73,81]. Furthermore, for patches with specific structures, such as MN patches, mechanical strength is also an important consideration.

In contrast, invasive patches, which adhere to internal organs, require strong adhesiveness to remain securely attached, ensuring effective treatment [82]. Since invasive patches are not easily removable, they must be capable of biodegrading *in vivo*, leaving minimal residue within the body [83,84].

### 2.3 Performances

Polymer patches serve a critical role in drug delivery, targeting the administration of small-molecule chemical drugs [85], biological macromolecules [86], and cells [87]. To optimize treatment efficacy and prolong the functionality of these patches, particularly for bioactive components, specific design principles must be carefully considered.

For small-molecule chemical drugs, which exhibit excellent permeability, polymer patches should be fabricated using materials with high molecular weight and mechanical strength to ensure slow, sustained drug release, crucial for long-term use [88,89]. Additionally, the poor aqueous solubility of some drugs necessitates the inclusion of water-miscible organic solvents, such as PEG 400, to form stable drug reservoirs within the patches [90].

Biological macromolecules are increasingly utilized due to their safety, target specificity, and favorable pharmacokinetics. However, their application is often limited by poor stability. Thus, selecting appropriate materials and formulations to preserve stability is essential. Key considerations include manufacturing and storage temperatures, drying conditions, and polymer concentration. Typically, low manufacturing temperatures, mild drying conditions, and optimized polymer concentrations help maintain drug activity [91,92]. The incorporation of stabilizing agents, such as trehalose, can further extend the active life of these drugs [93,94].

In cell therapy, polymer patches present unique challenges. One significant issue is the effective loading of cells, often constrained by the patch's structure [95]. This challenge may be addressed by integrating the delivery system with auxiliary devices, such as cell reservoirs or external actuators [87]. Another concern is maintaining an environment conducive to cell survival and therapy, given the difficulties associated with cell growth and transport [96,97]. The use of protective agents and supplementary nutrients can enhance cell viability. Moreover, factors like temperature, oxygen levels, bacterial presence, and other environmental conditions must be meticulously controlled [98].

In conclusion, the design of biomedical polymer patches must adhere to these principles. The materials selected should exhibit excellent biocompatibility, and the properties of these materials should be optimized according to the specific application. For different drug loadings, factors such as manufacturing processes, stability, capacity, and environmental conditions within the patches must be carefully considered. As materials science and technology continue to advance, more sophisticated polymer patches will be developed to meet these stringent requirements and broaden their applications.

## 3 Physical stimuli-responsive principle

Polymer patches have been widely utilized in the biomedical field, but traditional treatment methods often rely solely on the inherent characteristics of the materials, such as their degradation or dissolution, without considering the specific application environment and conditions. This lack of control can result in suboptimal therapeutic effects or pose the risk of drug overdose. Consequently, precise and intelligent control of treatment is essential.

A promising approach to achieve this is the development of stimuli-responsive polymer patches. These patches can deliver targeted therapy by exploiting differences between the microenvironment of diseased tissue and normal tissue or by responding to external stimuli, thereby enhancing treatment specificity and reducing side effects [99,100]. Stimuli-responsive devices can be categorized into two types: those responsive to endogenous stimuli and those responsive to exogenous stimuli. Endogenous stimuli-responsive devices react to internal chemical changes such as pH, glucose levels, enzymes, and ROS. In contrast, exogenous stimuli-responsive devices respond to externally applied physical stimuli, including light, magnetic fields, mechanical forces, and electric currents.

While endogenous stimuli-responsive patches are advantageous due to their simplicity and requiring no external stimulus, their application is often limited by the insufficient internal response in certain conditions, such as pain, cardiovascular disease, or neurological disorders, as well as the lack of timely therapeutic response [36]. Therefore, exogenous stimuli-responsive patches hold greater potential for treating a broader range of clinical conditions. This section will focus on external physical stimuli-responsive patches and their physical control strategies. The patches discussed are summarized in Table 1.

**Table 1:** Polymer patches with physical control strategies.

Polymer patches	Physical control	Application	Ref.
MN	NIR light	Melanoma healing	[106]
MN	NIR light and electric field	Tumor healing	[107]
MN	NIR light and heat	Diabetes treating	[45]
MN	NIR light and heat	Skin cancer healing	[119]
MN	Magnetic field	Versatile macromolecules delivery	[114]
Hydrogel	Magnetic field	Tumor healing	[115]
MN	External heat	Diabetes treating	[141]
MN	Electric current	Psoriasis treating	[123]
Hydrogel Scaffold	Electric current	Cardiac regeneration	[142]
Electronic patch	Electric current	Tissue regeneration Wound healing	[124]
MN	Mechanical force	Drug delivery	[129]
Hydrogel	Mechanical force	Wound healing	[128]
Hydrogel	Ultrasound	Protein delivery	[143]
MN	Ultrasound	Wound healing	[135]
Hydrogel	Humidity	Actuator	[139]
Hydrogel	Humidity	Humidity sensor	[140]

### 3.1 Light

Due to its non-invasive nature, high resolution, and time-controlled capabilities, light has become an attractive external stimulus for designing smart biomedical devices with improved spatiotemporal control over biomaterial behavior and the dynamic modulation of their properties [101,102]. To create light-responsive polymeric materials, photosensitive or thermal agents, such as nanoparticles, are typically encapsulated within polymers. Common light sources for this response system include near-infrared (NIR), ultraviolet (UV), and visible light. Among these, NIR is the preferred light for treatment because of its low ionizing potential and superior tissue penetration without causing damage [103,104]. As for the response principle, numerous patches with photothermal agents generate localized heat under NIR irradiation, promoting polymer degradation and drug release. The treatment process can be precisely controlled by adjusting the light intensity and irradiation duration [105].

**Fig. 3:**
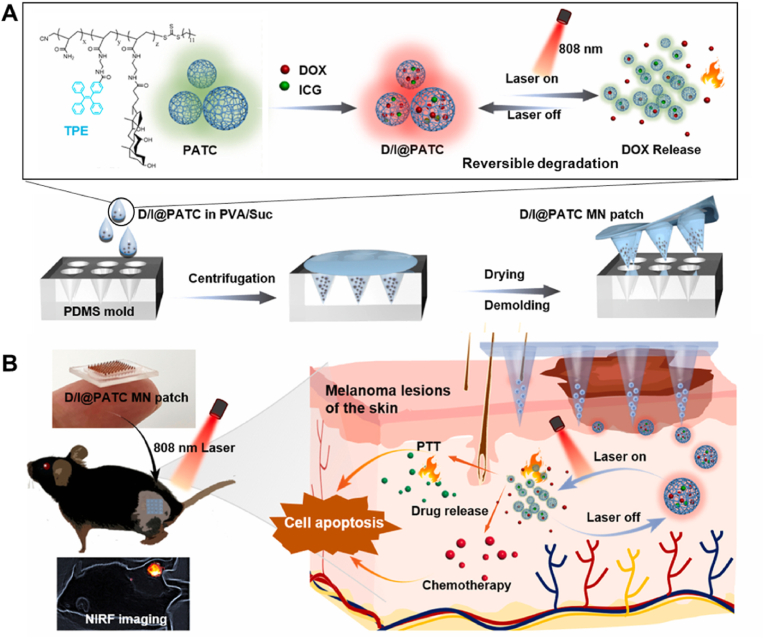
Mechanism of NIR light-responsive patch. A) The fabrication of photothermal conversion material and polymer patch. The drug DOX and the photothermal agent ICG were encapsulated in the PTAC particles. Under NIR irradiation, PTAC disassociated and released DOX and ICG for the tumor therapy. The process of disassociation was reversible. B) Therapy process of polymer patch. After applying the patch, NIR light could be used to control the release of DOX and ICG. Then, DOX was used as a drug for chemotherapy, and ICG was used as a photothermal agent for PTT. Two kinds of treatment were combined to treat melanoma. Reproduced with permission [106]. Copyright 2023, KEAI PUBLISHING LTD.

Light-responsive polymer patches enable controllable drug delivery. For instance, Wang et al. developed a self-monitoring microneedle (MN)-based delivery system (D/I@PATC MN patch) for melanoma treatment, integrating a dissolving MN patch with aggregation-induced emission (AIE)-active PATC microparticles [106]. The drug DOX and the photothermal agent ICG were encapsulated in PATC polymeric particles (Fig. 3A). Upon applying an 880 nm laser, PATC gradually dissociated, releasing DOX for chemotherapy and ICG for PTT to treat melanoma (Fig. 3B). In a tumor-bearing mouse model, one MN patch combined with two cycles of light irradiation demonstrated excellent controllable chemo and photothermal efficacy, with approximately 97 % melanoma inhibition and no evident systemic toxicity. This study highlights the potential application of polymeric devices with light control for clinical skin cancer treatment. Similarly, Gangrade et al. developed a photo-electroactive nanocomposite silk-based DDS loaded with DOX for tumor treatment [107]. This system allows on-demand drug release controlled by both NIR irradiation and an electric field, marking the first report of the simultaneous application of an electric field and NIR laser *in vivo* for localized tumor therapy, suggesting high clinical translational potential.

Although light responsiveness has been extensively studied, penetration depth limits applications in deep tissue, particularly for UV and visible light. A potential strategy involves converting incoming NIR light into higher energy UV–visible light to participate in photochemical reactions *in vivo* [37,108,109]. Moreover, during the light-triggered molecule delivery process in phototherapy, the uncontrolled burst release of drugs from nanocarriers can lead to short-term toxicity, while slow and incomplete metabolism of nanomaterials may result in long-term toxicity. Thus, material design requires further investigation [110].

### 3.2 Magnetic field

The magnetic field serves as an attractive external stimulus due to its potential to act as a remote switch, triggering on-demand responses. Additionally, its biocompatible and non-invasive nature enhances the application value of magnetic field-responsive polymeric patches [111]. These patches can be fabricated by incorporating metal-based nanoparticles (e.g., iron, cobalt, or nickel) into the polymers, which are intrinsically responsive to magnetic fields [112,113].

**Fig. 4:**
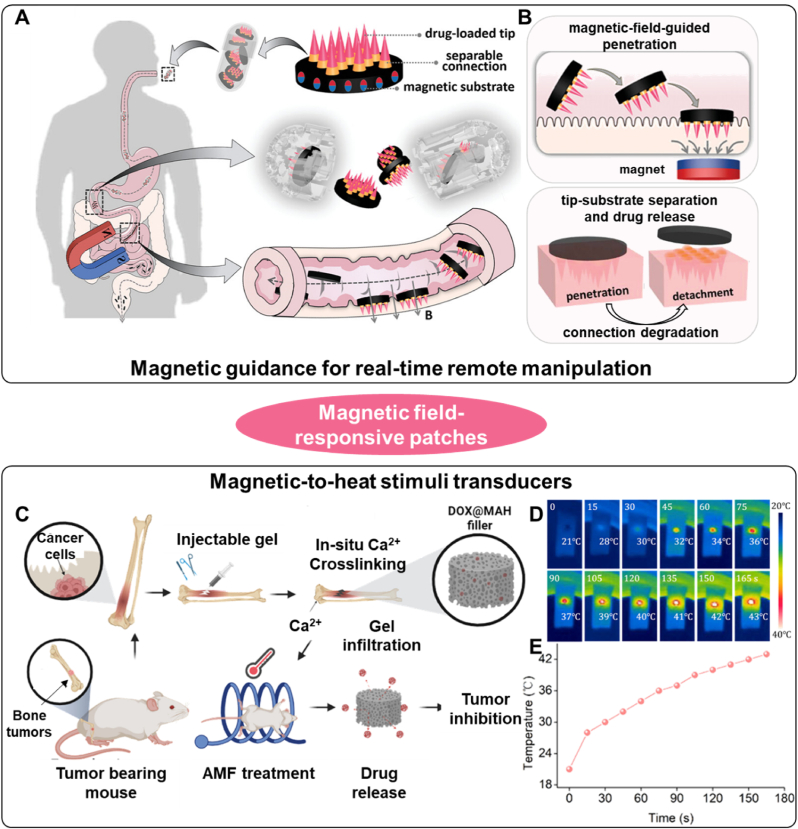
Two kinds of magnetic field response principles of polymer patches. A) MN patches are encapsulated in enteric capsules. Under the guidance of a magnetic field, the patches could reach the small intestine. B) The patch could penetrate small intestine walls under the guidance of magnetic fields. The tip would separate from the substrate for drug release after the degradation of the separable connections. Reproduced with permission [114]. Copyright 2021, WILEY-V C H VERLAG GMBH. C) Schematic illustration of Dox@MAH-mediated hyperthermia chemotherapy for bone tumor treatment. D) Infrared thermal images of a mold containing MAH under an alternating magnetic field. E) The temperature rising curve of hydrogel patch under magnetic field. Reproduced with permission [115]. Copyright 2024, AMER CHEMICAL SOC.

Two control modes have been developed for healthcare applications: (1) magnetic guidance for real-time remote manipulation of patches and (2) magnetic-to-heat stimulus transducers. For example, Zhang et al. introduced novel magneto-responsive microneedle robots for the efficient oral delivery of versatile macromolecules [114]. Under a specific magnetic field, these robots can overcome barriers and insert into tissue for drug delivery due to their polarized magnetic substrate (Fig. 4A and B). The robots were used to deliver insulin and regulate blood glucose levels in pigs, demonstrating their practical application. Regarding magneto-thermal conversion, Hu and colleagues developed a DOX-loaded magnetic alginate hydrogel (DOX@MAH) for synergistic bone tumor hyperthermia chemotherapy (Fig. 4C) [115]. Iron oxide nanoparticles served as magnetic hyperthermia agents to generate heat for tumor elimination (Fig. 4D and E). Combined with DOX for chemotherapy, this system demonstrated high efficacy in eliminating residual tumor tissue and represented a highly effective method for treating bone tumors.

Despite the advantages of remote control, there are some problems that remain to be solved for the application of magnetic field-responsive patches, especially invasive patches. Due to the complex physical and chemical environment *in vivo*, the remote control of patches could be influenced by many factors, including blood flow and pH levels [116,117]. The structure of patches and materials to withstand corrosion needs further study.

### 3.3 Temperature

Many polymers exhibit temperature-sensitive properties and can undergo phase changes at specific temperatures. Thus, when external thermal stimuli are applied, the resulting temperature change can alter the polymer structure and facilitate the release of drugs within the patches [118].

**Fig. 5:**
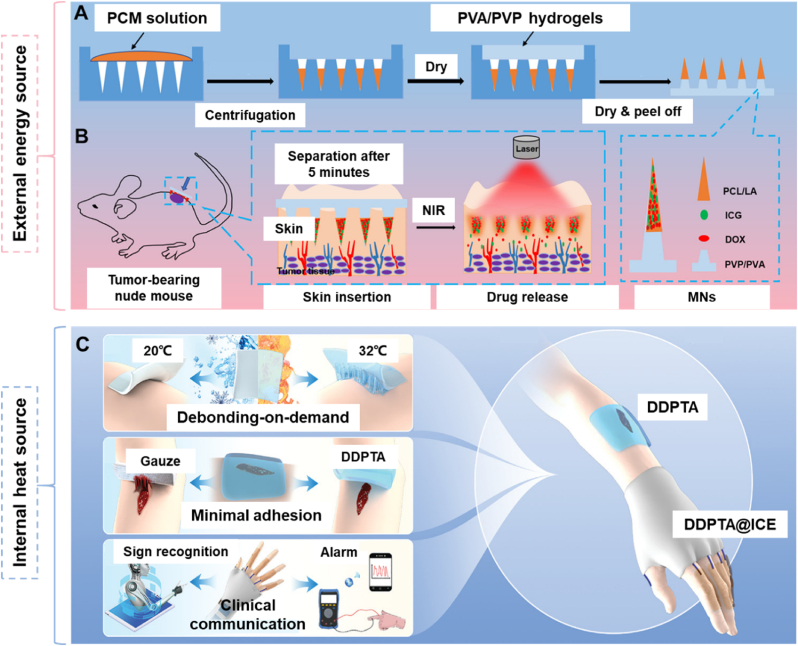
Working principles of two kinds of thermo-responsive polymer patches. A) Fabrication process of the thermo-responsive patch. B) Delivery process of DOX for tumor healing. ICG worked as an internal heat source under NIR irradiation to control the process of drug delivery. Reproduced with permission [119]. Copyright 2020, AMER CHEMICAL SOC. C) The advantages of a thermo-responsive patch, including temperature-response debonding-on-demand, wound sealing with minimal adhesion, sign language recognition, and remote clinical communication. Reproduced with permission [120]. Copyright 2022, WILEY.

Numerous studies have utilized photothermal converters as heat sources for treatment. For example, Liu's group fabricated NIR light-responsive polymer MNs for diabetes treatment [45]. When these MNs were irradiated with NIR light, the light-to-heat transduction induced by Bi nanodots caused the MNs to dissolve, releasing metformin to treat diabetes. Gao et al. designed a separable MN patch for skin tumor therapy [119]. Upon NIR irradiation, the photothermal conversion of ICG ablated the MNs, releasing DOX and treating the tumor (Fig. 5B).

Apart from external heat sources, endogenous heat from body tissues can also be utilized for controllable treatment. For instance, Zeng et al. developed a polymeric wound patch for minimal adhesion [120]. This patch integrates a DDPTA with an ICE. Due to the semicrystalline-to-amorphous transition triggered by temperature changes, the patch remains soft and adherent at skin temperature but becomes hard and non-tacky when cooled (Fig. 5C), allowing for unique temperature-triggered quick adhesion and non-forced detachment from the skin. These characteristics enable the patch to avoid the significant drawbacks of hydrophilic adhesives.

Temperature-responsive patches often require additional control strategies, such as light and electricity, to regulate heat generation. Consequently, complex agents and devices are necessary during the treatment process, limiting their application in daily therapy. Moreover, the generated heat may inactivate drugs, particularly macromolecular drugs. Further investigation and design are needed for low-temperature-responsive patches.

### 3.4 Electric current

Electric current-responsive polymeric patches are primarily prepared by using conductive polymers such as polyaniline, poly (N-vinyl imidazole), and PPy, or by combining them with conductive nanoparticles [121,122]. Upon the application of electric current, these polymers can undergo reversible redox reactions, altering their structure due to electron transport.

**Fig. 6:**
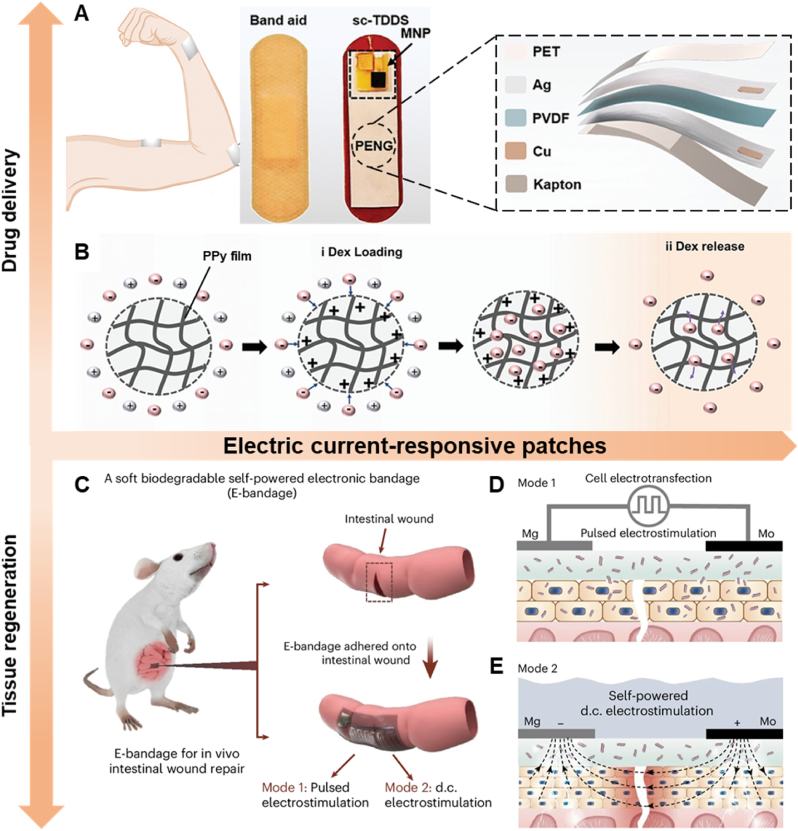
Treatment mechanism of electric current-responsive patches. A) Structure of patch and PENG. B) Mechanism of controlled delivery of Dex. Dex was directly doped into the PPy backbone to achieve the drug loading when the PPy film was oxidized and deposited. Dex was de-doped from PPy to realize the drug release when the PPy film was reduced at a certain voltage. Reproduced with permission [123]. Copyright 2021, WILEY-V C H VERLAG GMBH C) Illustration of applying the dual-electrostimulation E-bandage onto an intestinal wound. D), E) Dual electrostimulation operated by the Mo–Mg interdigital microelectrode pair. Mode 1: ET of local intestinal epithelial cells for the delivery of EGF-encoded DNA plasmids. Mode 2: ES to the local intestinal epithelial cells for enhancing secretion of healing factors. Reproduced with permission [124]. Copyright 2024, NATURE PORTFOLIO.

Electric current can be employed to control the release of drugs from polymers. For instance, Yang et al. developed a self-powered electro-responsive MN patch for on-demand transdermal delivery of Dex to treat psoriasis [123]. In this system, a PENG generates electric current from biomechanical motions such as bending, breathing, and heartbeat (Fig. 6A). Under a certain voltage, the hydrogel can load or release Dex for treatment (Fig. 6B). In vivo studies on a psoriasis-bearing mouse model demonstrated that MNs with electric stimulation yielded better results than those without electric stimulation. This research highlights the potential of electric current-responsive polymers for drug delivery.

Beyond drug delivery, conductive polymers can also be used to fabricate bio-functional tissues, such as cardiac tissues. Esmaeili et al. recently developed hydrogel scaffolds incorporating gold and silica nanoparticles to enhance cardiac function and regeneration [123]. The electrical conductivity of these hydrogel scaffolds significantly influenced tissue signal propagation, improving the functionality and structure of cardiac tissue to some extent. However, electrical conductivity may not be the sole factor affecting cardiac tissue, necessitating further studies to explore additional influencing factors. Similarly, Wu et al. created a self-powered electronic patch for wound healing [124]. This patch included an Mg/Mo galvanic cell to provide electric current. Two types of electrostimulations, ET and direct current ES, were used to activate and enhance the proliferation of neighboring epithelial cells (Fig. 6C). ET promoted the expression of healing factors, while ES increased the secretion of healing factors in the transfected cells (Fig. 6D and E). This study presents a promising strategy for wound healing.

Electric current-responsive patches typically require auxiliary equipment to provide energy for treatment, significantly limiting their application [123,124]. Although some self-powered patches have been developed, the operational duration of these patches requires further investigation. Additionally, the use of certain metal materials may impact the biocompatibility of the patches. Further exploration is needed to advance the application of these polymer patches.

### 3.5 Mechanical force

Mechanical force-responsive patches can respond to mechanical strains from muscles, tendons, bone joints, and other organs. When forces are applied to the patches, the polymer structure changes, allowing controlled drug release. Unlike other exogenous stimuli-responsive devices, they do not require additional equipment to control the treatment process, making them ideal for simple treatments [125,126]. Treatment control can be achieved through self-induced stretching, bending, pressing, or insertion without concern for the drug's structure (anionic/cationic/large molecules/thermal-sensitive drugs) [127]. Thus, mechanical force-responsive devices are a promising strategy for self-administered treatments.

**Fig. 7:**
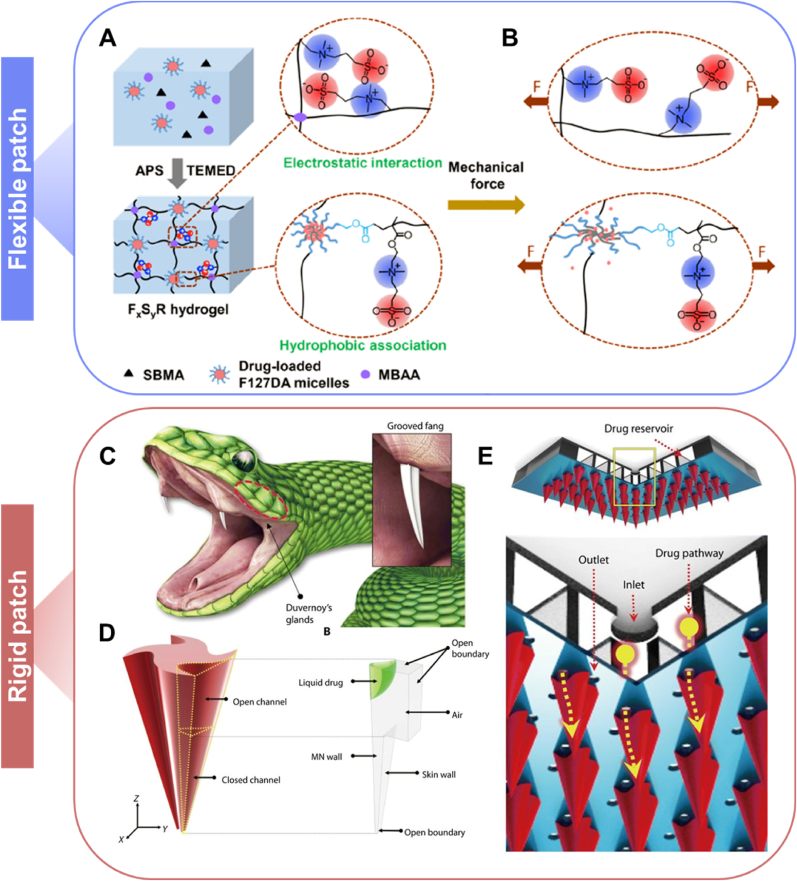
Two kinds of mechanical force-responsive patches. A) Preparation of hydrogel patch. B) Deformation of hydrogel network and drug release under mechanical force. Reproduced with permission [128]. Copyright 2020, AMER CHEMICAL SOC. C) Venom delivery system of snake. Venom could run along the fang and rapidly flow. D) Structure of MNs, having upper wide and lower narrow channels. E) Conceptual diagrams of the patch. Liquid drugs were loaded into the reservoir. Under a pressure from a thumb, the drugs could roll down the grooves and enter the skin for drug delivery. Reproduced with permission [129]. Copyright 2019, AMER ASSOC ADVANCEMENT SCIENCE.

Mechanical force-responsive patches are typically flexible. Fang et al. designed a mechano-responsive, tough, and antibacterial zwitterionic hydrogel for wound healing [128]. This hydrogel prevents protein adsorption and bacterial adhesion, and antibiotic delivery can be controlled by the extent and cycles of mechanical loading and unloading (Fig. 7B). This hydrogel shows great potential as a wound dressing for acute wounds subject to frequent movements. Rigid patches can also be mechanical force-responsive. For example, Bae group developed a MN patch that mimics the grooved fangs of rear-fanged snakes for liquid formulation delivery (Fig. 7C) [129]. This patch requires only gentle thumb pressure to trigger liquid-phase drug and vaccine delivery in seconds, similar to snake fangs (Fig. 7D and E). The results demonstrated that administering ovalbumin and the influenza virus with the microneedle patch induced robust antibody production and a protective immune response in guinea pigs and mice, highlighting its application value.

Mechanical force-responsive patches are suitable for on-demand drug delivery, particularly for wound healing. However, users may find it difficult to control the external forces, potentially affecting treatment outcomes. Further studies are needed for precise control of drug delivery. Additionally, the challenge of using mechanical force-responsive patches for continuous treatment remains to be addressed.

### 3.6 Ultrasound

Ultrasound is widely used in the biomedical field due to its safety and non-invasive nature [130]. It can penetrate human skin and connective tissue to depths exceeding 5 cm [131]. Therefore, ultrasound waves are effective for remotely controlled drug delivery, particularly *in vivo* [131].

**Fig. 8:**
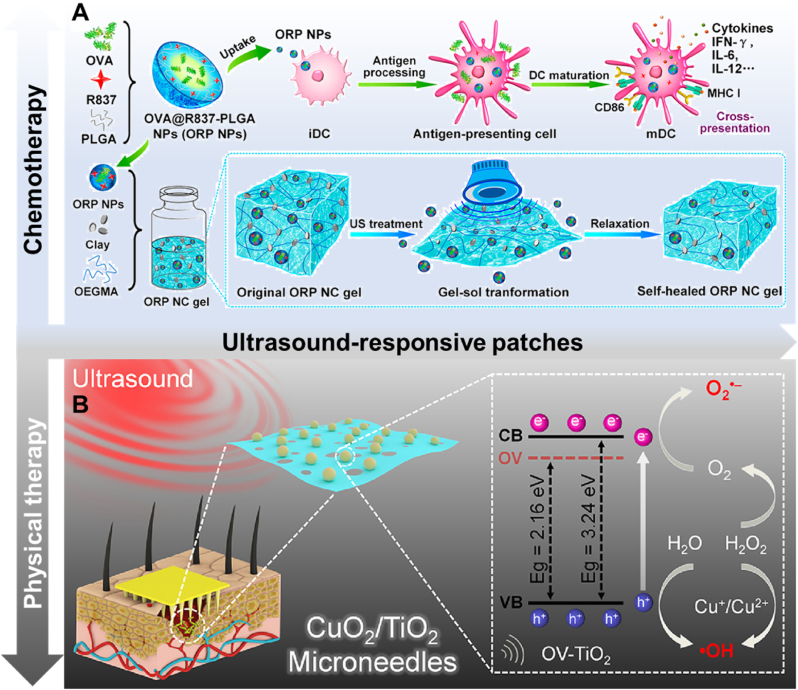
Two kinds of ultrasound-responsive patches. A) The fabrication process of a hydrogel loaded with nano-vaccines and the drug delivery process triggered by the ultrasound. Reproduced with permission [132]. Copyright 2021, AMER CHEMICAL SOC. B) Brief illustration of the mechanism of sonochemodynamic and sonothermal therapy. The combination of CuO_2_ nanoclusters and OV-TiO_2_ nanosheets could transfer the energy of ultrasound to heat for thermal therapy and provide ROS for chemo-dynamic therapy. Reproduced with permission [135]. Copyright 2023, ELSEVIER SCI LTD.

The acoustic energy from ultrasound stimulation can disrupt the structure of some polymers and promote their disassembly, facilitating drug delivery. For example, Meng et al. designed an ultrasound-responsive, self-healing hydrogel system loaded with nano-vaccines for cancer immunotherapy [132]. This hydrogel transforms into a sol state upon ultrasound treatment, allowing a burst release of vaccines, and self-heals back into a gel in the absence of ultrasound (Fig. 8A). Combined with immune checkpoint blockade, the hydrogel can prevent postoperative tumor recurrence and metastases.

Beyond drug delivery, ultrasound can be utilized for physical therapy. Ultrasound-responsive materials, such as acoustic sensitizers, can produce ROS upon ultrasound excitation [133]. Additionally, some materials can convert sound energy into heat for sonothermal therapy [134]. Liang et al. developed ultrasound-activatable microneedles for antibacterial therapy [135]. They created a unique CuO_2_/TiO_2_ heterostructure composed of ultrasmall (CuO_2_) nanoclusters and sonosensitized ultrathin OV-TiO_2_ nanosheets, which were loaded into the patch. Under ultrasound waves, this structure generates ROS to kill bacteria (Fig. 8B). Additionally, the ultrasound energy is converted into heat, enabling sonothermal antibacterial therapy. The elimination rate against multidrug-resistant pathogens was over 99.9 % within 5 min, both *in vitro* and *in vivo*, demonstrating the potential of sound therapy for wound healing.

Ultrasound waves have shown potential as a control method for therapy, particularly in wound healing. However, responsive patches must be paired with ultrasound-generating machines, limiting their daily use. Smaller and simpler ultrasound sources need to be developed.

### 3.7 Humidity

Humidity is a crucial indicator of human physiological information [136]. It can also be utilized as a physical stimulus for biomedical applications. Humidity-responsive patches are typically prepared using hydrogels, which exhibit excellent biocompatibility, elasticity, and responsiveness to humidity [137,138]. When water droplets or vapor are absorbed, hydrogel networks expand due to the diffusion of water molecules between cross-linked structures. Conversely, the networks undergo volumetric contraction when they lose water [53]. These structural changes can be harnessed for biomedical applications. Additionally, the absorption and loss of water within the hydrogel can affect the transport of water molecules and ions, which is linked to the hydrogel's conductivity.

**Fig. 9:**
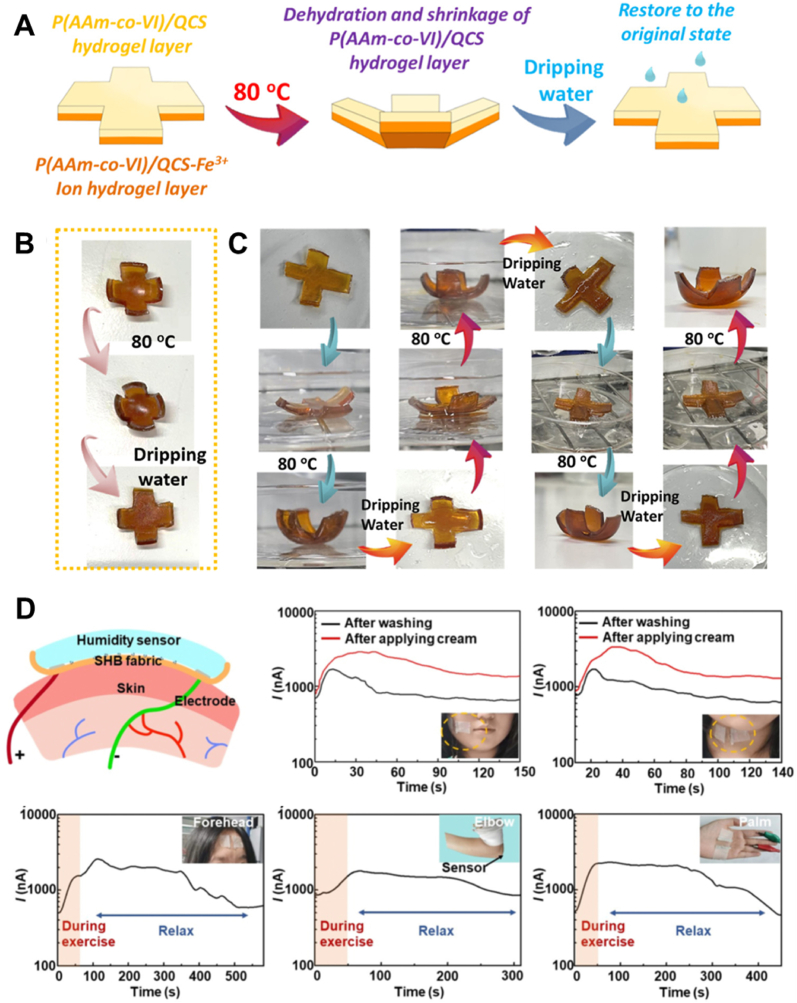
Humidity responsive patches. A) Schematic process of actuation of hydrogel humidity actuator. B) Actuation process of hydrogel actuator. C) Four temperature/humidity actuation cycles of the hydrogel actuator. Reproduced with permission [139]. Copyright 2022, AMER CHEMICAL SOC D) Real-time monitoring of skin humidity at different parts of the body using the hydrogel sensor, including cheek, neck, forehead, elbow and palm. Reproduced with permission [140]. Copyright 2023, ROYAL SOC CHEMISTRY.

This property can be exploited for humidity-responsive actuators, which have potential applications in artificial muscles and smart robotics. For example, He et al. developed a humidity-responsive hydrogel actuator based on P (AAm-co-VI)/QCS-Fe^3+^ ionic hydrogel and P (AAm-co-VI)/QCS hydrogel [139]. In environments with high temperature or low humidity, the different rates of water loss in the two types of hydrogels induce structural changes, driving the actuation process (Fig. 9A). The patch can return to its original state after water is applied (Fig. 9B). Besides actuators, humidity-responsive patches can also function as humidity sensors due to changes in conductivity in different humidity environments. Pan et al. designed a fast-response, waterproof, wearable humidity sensor based on an ultrathin hierarchical hydrogel–carbon nanocomposite [140]. Water molecules are adsorbed on the nanocomposite surface via hydrogen bonds. At high relative humidity, a hydration layer forms, facilitating the free transport of water molecules and ions, which significantly enhances conductivity. The hierarchical surface topography increases the specific surface area, endowing the sensor with high sensitivity and a wide humidity range.

Although humidity-responsive hydrogels show great potential for biomedical applications, balancing actuator and sensor sensitivity with mechanical strength remains challenging. Many hydrogel patches are made with thick hydrogels to ensure strength. However, high sensitivity relies on the diffusion of water molecules within the hydrogel, which can be impeded by large thickness. Further research is needed to develop hydrogel patches with low thickness and high strength.

## 4 Fabrication methods of polymer patches

**Fig. 10:**
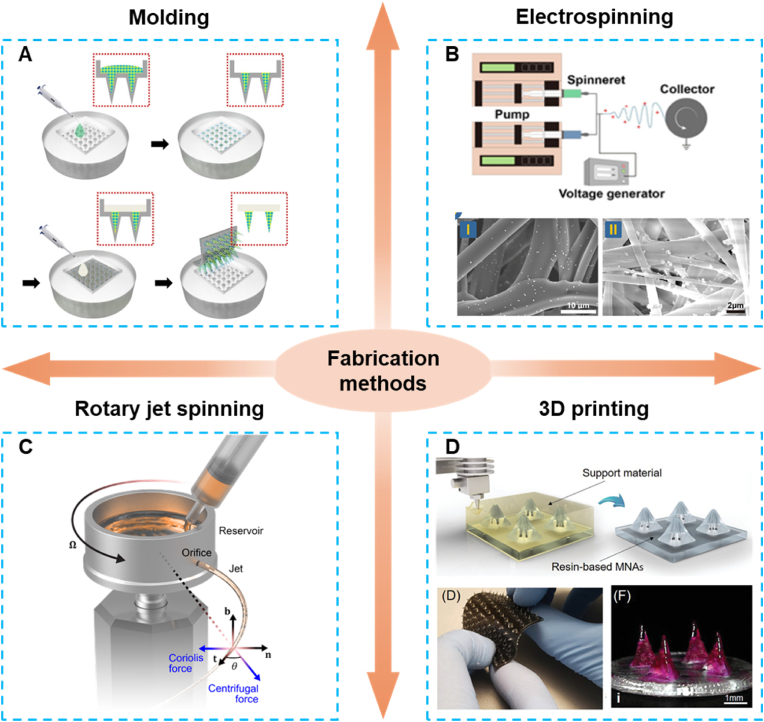
Fabrication methods of polymer patches. A) Molding method of manufacturing polymer patches. Reproduced with permission [144]. Copyright 2023, AMER ASSOC ADVANCEMENT SCIENCE. B) Electrospinning for generating patches from nanofibers. Reproduced with permission [145]. Copyright 2023, ELSEVIER. C) Rotary jet spinning for the fabrication of patches. Reproduced with permission [146]. Copyright 2018, ELSEVIER. D) 3D printing for manufacturing polymer patches. Reproduced with permission [147]. Copyright 2020, WILEY-V C H VERLAG GMBH.

In recent years, researchers have employed various methods to manufacture a wide range of polymer patches. When designing a polymer patch, the primary objective must be determined first, as it typically dictates the choice of materials and structure. Subsequently, factors such as drug type and loading dose, desired pharmacokinetics/pharmacodynamics, and target application must be carefully considered. This section summarizes the various fabrication methods reported for polymer patches, which are illustrated in Fig. 10, with a comparative analysis provided in Table 2.

**Table 2:** Comparison of different fabrication methods of polymer patches.

Fabrication method	Mechanism	Advantages	Limitations
Molding	Cast polymers into molds and solidify them to form specific structure	· Widely used · Molds can be used repeatedly · Quick fabrication · Cheap method	· Cured air bubbles can alter design. · Multi-step process · Require master mold
Electrospinning	Use the electrostatic repulsion of the polymer solution to produce polymeric fibers	· Simple and low-cost equipment · Nano-micro scale fibrous structure · High surface-to-volume ratio, high porosity, and variable pore size distribution	· Requires conductive polymers and solvents · Low production rate · Probability of remaining solvent · Rely on high voltage for fiber formation
Rotary jet spinning	Use centrifugal forces to extrude nanofibers from solution	· Control on fiber morphology · Fabrication at the nano to micro-scale · High production rate · Room temperature processing	· Probability of remaining solvent · Low porosity · Require post-processing
3D printing	Convert digital images into a physical 3D model with the deposition of material layers	· Good printing resolution · Customize patches with various structures	· Long fabrication period · Complex preparation process · Require photosensitive or thermosensitive materials · Using high temperature or UV light

### 4.1 Molding

Molding is the most widely used and accepted method for fabricating polymer patches [148]. This technique involves using molds with specific structures to create polymer patches for various applications. The process includes preparing molds, casting polymers onto or into the molds, removing bubbles by centrifugation or vacuum, solidifying the material through drying or photo-crosslinking, and finally, detaching the patches from the molds. The molds can be reused to produce multiple patches [149]. Additionally, patch parameters can be optimized by adjusting the mold design [150]. Due to its cost-effectiveness, good reproducibility, and scalability, molding has significant potential for large-scale patch production.

Various materials, such as PVA [151], PDMS [152] and acrylic [153] can be used to fabricate molds. PDMS, in particular, is favored for small-scale molding due to its excellent transcription ability, thermal stability, and low adhesion, which facilitates the easy separation of patches from the mold. Moreover, molds can be easily reproduced from a single template, enhancing production efficiency [154].

However, despite its advantages in the biomedical field, molding presents some challenges. The multiple fabrication steps are time-consuming and may lead to a loss of activity in encapsulated drugs. Additionally, the use of heat or UV light during solidification restricts the use of sensitive drugs, such as peptides, proteins, and vaccines [155].

### 4.2 Electrospinning

Electrospinning is a straightforward and cost-effective technique that utilizes electrostatic repulsion in a polymer solution to produce polymeric fibers [156,157]. It has been widely employed in fabricating patches with micro- and nanofibrous structures [158]. The fabrication process involves dissolving polymers in a specific solvent and loading the solution into a capillary tube for electrospinning. An electric charge is then applied to the surface of the polymer solution. Under the influence of the electric field, the repulsive electric forces overcome the surface tension, causing the charged jet to exit the tip of the Taylor cone, forming a jet in the space. As the solvent evaporates, the polymer patches are formed [159].

Despite the advantages of electrospinning in fabricating polymer patches, certain limitations constrain its application. The primary drawback is the limited control over the pore structure of the fibers, which is mainly determined by the fiber diameter. Smaller average pore sizes can reduce cellular infiltration in polymer patches [160]. Additionally, the use of toxic solvents may negatively impact the biocompatibility of the polymer patches [161].

### 4.3 Rotary jet spinning

RJS is a cost-effective, environmentally friendly method for fabricating polymeric nanofibrous structures [162]. Unlike electrospinning, RJS does not require the induction of an electric charge into the polymer solution. Instead, it utilizes the centrifugal forces generated by a rotating reservoir with single or multiple micron-scale orifices to extrude nanofibers circumferentially. The properties of the resulting fibers, such as diameter and alignment, can be easily controlled by adjusting the polymer solution's characteristics and spinning parameters, including solution viscosity, rotation rate, and extrusion speed [163]. However, the primary challenge of RJS lies in optimizing the polymer solution properties (elasticity and surface tension), managing solvent fluctuations, and fine-tuning parameters like capillary diameter and collecting radius to produce ultra-fine fibers without jet rupture or droplet formation [164].

### 4.4 3D printing

3D printing is an advanced additive manufacturing technology that enables rapid prototyping at a low cost and with high throughput [165]. This technique converts digital designs into physical 3D models by depositing material in layers [166]. Unlike other methods, 3D printing offers the advantage of customizing intricate structures and creating personalized shapes [166,167]. Additionally, it is considered a green, intelligent manufacturing technology with low production costs [168,169].

Based on the fabrication process, 3D printing can be categorized into two types: point-by-point and layer-by-layer [170,171]. In point-by-point printing, the polymer patch structure is formed through polymerization or deposition of polymers at specific points [172]. In layer-by-layer printing, photosensitive polymers are selectively cured through light-activated polymerization, layer by layer [172]. This process allows for precise control over the formation of specific polymer patch structures.

However, despite its advantages, 3D printing faces several challenges. While it allows for the creation of high-quality, customized patches, the process is relatively slow. Moreover, the technology requires the use of photosensitive or thermosensitive materials, limiting the range of compatible materials. Additionally, the need for UV light or high temperatures during manufacturing restricts its application with sensitive drugs, such as peptides, proteins, and vaccines.

## 5 Therapeutic applications with physical stimuli

Advancements in materials technology have led to the development of various polymer patches for healthcare applications, including nervous system restoration, heart therapy, diabetes management, wound healing, cancer treatment, obesity therapy, hair growth and scar therapy. Additionally, these polymer patches are often integrated with physical control mechanisms to enhance therapeutic outcomes. This section will discuss the therapeutic applications of physical stimulus-responsive polymeric patches. Table 3 provides a summary of the types of patches, the physical stimuli they respond to, their biomedical applications, and specific treatment methods, offering valuable references for future research.

**Table 3:** Application of physical stimulus-responsive polymer patches.

Polymer patches	Physical stimulus	Application	Treatment method	Ref.
Fiber	NIR light	Nervous system	Delivery of biological effectors	[178]
Hydrogel	NIR light	Nervous system	Repair injured nervous system	[179]
Scaffold	NIR light	Nervous system	Transduce light energy to acoustic and activate neurons to promote neural outgrowth	[180]
Scaffold	Ultrasound	Nervous system	Deliver ES to promote neural stem cell differentiation and endogenous angiogenesis	[181]
MN	NIR light	Heart therapy	Deliver VEGF for neovascularization	[191]
Photonic patch	Light	Heart therapy	Generate oxygen for treatment of hypoxia diseases	[192]
Hydrogel	NIR light	Heart therapy	Deliver copper-ion and thermal therapy	[194]
Scaffold	NIR light	Heart therapy	Attach to the heart and repair it	[195]
Active patch	Magnetic field	Heart therapy	Provide mechanical circulatory to assist cardiac circulation	[196]
MN	NIR light	Diabetes healing	Deliver metformin	[206]
MN	NIR light	Diabetes healing	Deliver metformin	[207]
MN	Mechanical force	Diabetes healing	Deliver insulin	[49]
Hydrogel	Thermo	Wound healing	Deliver DS	[213]
Hydrogel	Thermo	Wound healing	Deliver DS and bFGF	[214]
Nanofibrous membrane	NIR light	Wound healing	PTT and deliver BBH	[217]
Hydrogel	Light	Wound healing	PTT and PDT	[218]
Hydrogel	NIR light	Wound healing	PTT and PDT	[219]
Hydrogel	Thermo	Cancer healing	Deliver MTX	[223]
Hydrogel	Thermo	Cancer healing	Deliver heparin	[224]
MN	NIR light	Cancer healing	Deliver thrombin and temozolomide	[226]
Hydrogel	NIR light	Cancer healing	PTT	[227]
MN	NIR light	Cancer healing	PDT and deliver CDDP	[228]
MN	NIR light	Obesity treatment	Deliver Rosi	[244]
MN	NIR light	Obesity treatment	Deliver Rosi	[245]
MN	NIR light	Obesity treatment	PTT and deliver mirabegron	[247]
Hydrogel	NIR light	Obesity treatment	PTT and deliver mirabegron	[248]
Hydrogel	Magnetic field	Obesity treatment	MHT	[263]
MN	Magnetic field	Hair growth	Deliver Mx	[253]
Hydrogel	Thermo	Hair growth	Deliver JAM-A protein	[254]
Hydrogel	635 nm light	Scar therapy	PDT	[258]
Stacked electret	Electric field	Scar therapy	Electrical stimulation	[259]
MN	Mechanical force	Growth hormone deficiency treating	Deliver rhGH	[260]
MN	Mechanical force	Contraception	Deliver levonorgestrel	[261]
MN	NIR light	systemic lupus erythematosus therapy	Deliver interferon γ and dexamethasone	[262]

### 5.1 Nervous system

Repairing and regenerating injured nervous systems, including the brain, spinal cord, and peripheral nerves, remain significant challenges in the biomedical field [173,174]. One potential treatment strategy involves neural interfaces that use implanted conductive electrodes to restore or substitute neural functions [175]. However, surgical injuries and low spatial resolution limit their therapeutic efficacy [176]. Consequently, conductive polymer patches, which are more adaptable to the microenvironments of living tissues, have emerged as candidates for neural interfaces [177].

Polymer patches for nervous system repair can be categorized into two types: invasive and non-invasive. Invasive patches typically adhere directly to the injured nervous tissue for treatment. For example, Xue et al. developed a NIR-responsive polymer patch designed to release biological effectors promoting neurite outgrowth [178]. The patch comprised two layers of PCL fibers and microparticles of a PCM made from a eutectic mixture of lauric acid and stearic acid. Microparticles loaded with EGF were sandwiched between the two fiber layers. Upon irradiation with a NIR laser, the PCM melted, swiftly releasing the biological effectors, which promoted directional migration of NIH-3T3 fibroblasts and neurite outgrowth from PC12 cells. Under masked irradiation, the average and longest neurite lengths increased from 659 ± 114 μm and 799 ± 62 μm to 1093 ± 171 μm and 1269 ± 89 μm, respectively, confirming the acceleration of neurite outgrowth.

**Fig. 11:**
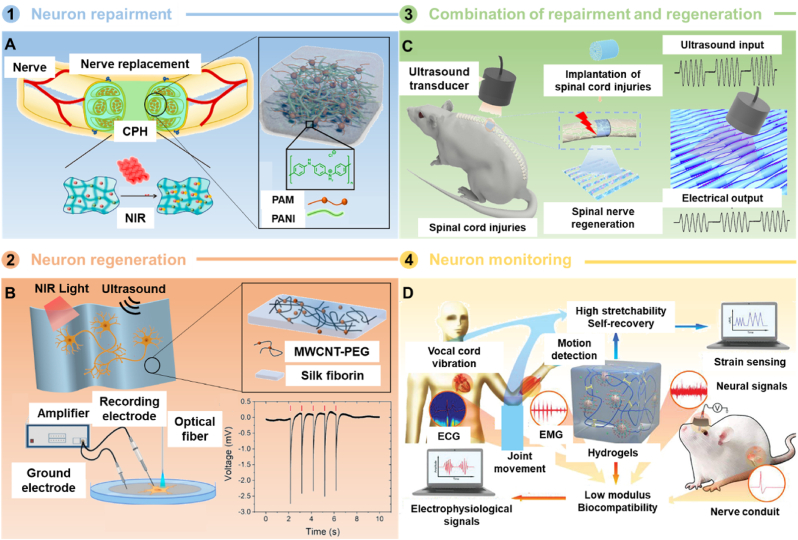
Application of polymer patches in the nervous system. A) NIR-responsive patches used for neuron repairment. Reproduced with permission [179]. Copyright 2020, AMER CHEMICAL SOC. B) NIR and ultrasound-responsive patches utilized for neuron regeneration. Reproduced with permission [180]. Copyright 2022, AMER CHEMICAL SOC. C) Ultrasound-responsive patches for regeneration and repairment of the nervous system. Reproduced with permission [181]. Copyright 2022, AMER CHEMICAL SOC. D) Conductive hydrogel for robust electroencephalogram signals recording on the hairy scalp. Reproduced with permission [77]. Copyright 2022, WILEY.

Beyond drug delivery, nonpharmacological methods employing physical stimuli have also been explored for nervous system repair. Dong et al. designed a light-stimuli-responsive and stretchable CPH for this purpose [179]. Under NIR light, the conductivity of the CPH was enhanced, promoting the conduction of bioelectrical signals (Fig. 11A). Moreover, when mechanically elongated, the CPH maintained high conductivity durability. After connecting the damaged sciatic nerve with the CPH, the maximum tension of the gastrocnemius muscle reached up to 1.7 g under an additional 0.1 V stimulus, showing no significant difference from the intact sciatic nerve. Zheng's group utilized silk fibroin loaded with PEG-functionalized CNTs to develop a polymer scaffold for neural regeneration [180]. The embedded CNTs could absorb pulsed NIR-II light and transduce light energy into acoustic energy, activating neurons to promote neural outgrowth (Fig. 11B). Compared with the unstimulated group, on-demand photoacoustic stimulation increased neurite outgrowth by 1.74-fold in a rat dorsal root ganglion model. Chen's group prepared a three-dimensional multichannel piezoelectric scaffold by electrospinning PLA nanofibers incorporated with biodegradable high-performance piezoelectric potassium sodium niobate (K_0.5_Na_0.5_NbO_3_, KNN) nanowires for spinal cord injury repair [181]. Using programmed ultrasound irradiation, the scaffold could deliver on-demand *in vivo* electrical stimulation, promoting neural stem cell differentiation and endogenous angiogenesis at the lesion site (Fig. 11C).

Non-invasive patches typically adhere to external tissues such as skin and mucosa. Currently, most are utilized for monitoring the nervous system. Li's group developed a polyvinyl alcohol/polyacrylamide double-network hydrogel electrode for robust electroencephalography recording on the hairy scalp [182]. The semi-dry electrode exhibited low contact impedance (18 ± 8.9 kΩ at 10 Hz), a small offset potential (0.46 mV), and negligible potential drift (1.5 ± 0.4 μV min^−1^). It conformed well to the hairy scalp and could accurately capture electroencephalogram signals. Xie et al. also employed hydrogel to develop a stable electrode for a wearable brain-computer interface [183]. The electrode could record electroencephalogram signals and was used in online BCI-based functional electrical stimulation for post-stroke rehabilitation. Liang's group designed a highly stretchable hydrogel as a wearable sensor [77]. The hydrogel exhibited good chemomechanical compatibility with tissues and minimal signal attenuation over extended periods, making it ideal for long-term implantable sensory devices (Fig. 11D).

Although polymer patches have demonstrated significant advantages in repairing and regenerating the nervous system, several challenges remain. For invasive patches, implantation *in vivo* is still required, making the process complex and limiting their application. Non-invasive patches, on the other hand, have not yet been utilized for treatment purposes. A potential approach is to combine non-invasive patches with non-invasive stimulation methods, including electrical and magnetic stimulation [184,185]. Such patches could monitor the status of the nervous system and generate deep stimulation for treatment in response to specific physical signals. These advancements may hold great potential for applications in nervous system healthcare.

### 5.2 Heart therapy

Cardiovascular diseases have become a global health crisis, with MI being a leading cause of death [186,187]. Traditional treatment options, including pharmaceutical therapy, medical device implantation, and organ transplantation, are limited by high invasiveness, donor organ scarcity, and immune rejection [188]. A novel and promising approach is the use of engineered cardiac patches, which offer a safer and more effective method for clinical MI treatment [189,190].

**Fig. 12:**
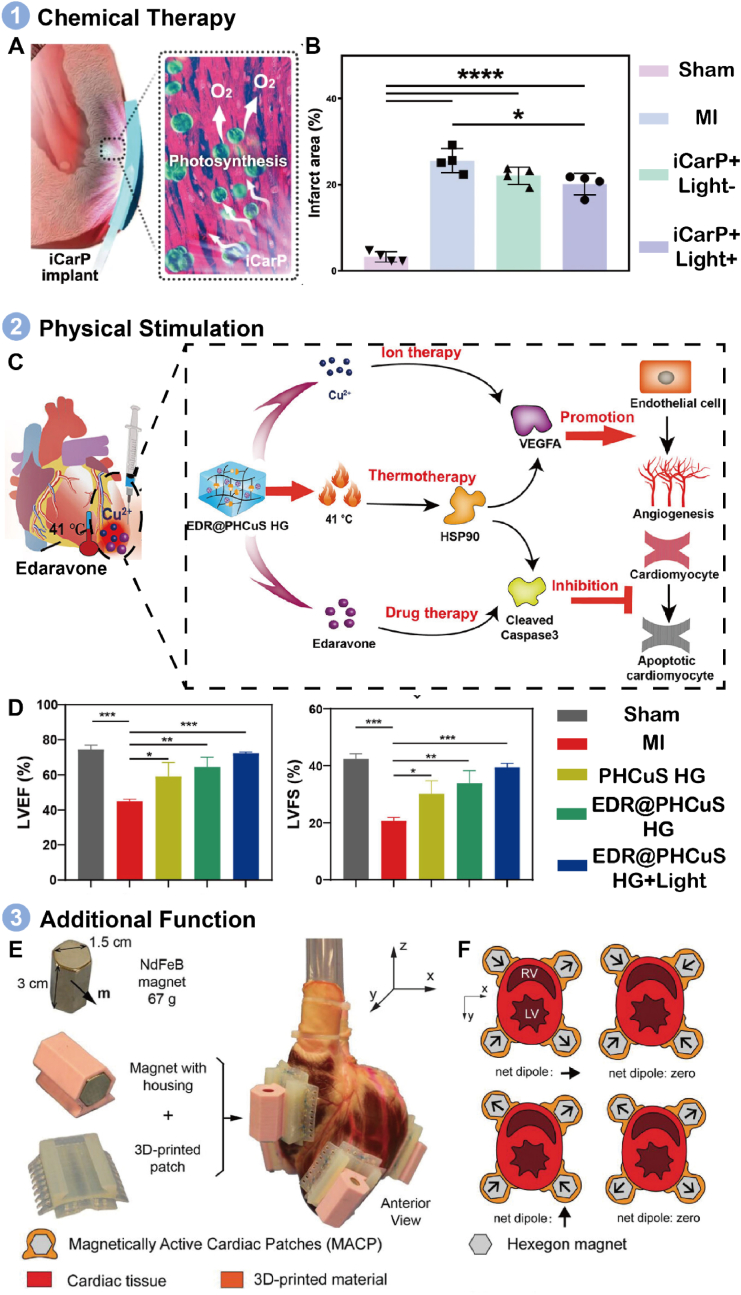
Application of polymer patches on heart therapy. A) Mechanism of treatment by using ICarP to generate green oxygen. B) Quantitative analysis of infarct area based on Masson's trichrome staining. Reproduced with permission [192]. Copyright 2023, NATURE PORTFOLIO. C) Therapy mechanism of NIR responsive hydrogel patch by combining thermotherapy and chemical therapy. D) Quantitative analysis of cardiac function parameters. Reproduced with permission [194]. Copyright 2023, AMER CHEMICAL SOC. E) Structure of MACP for heart therapy. F) Under certain magnetic fields, the patch could provide mechanical circulatory to assist cardiac circulation. Reproduced with permission [196]. Copyright 2021, WILEY.

Certain polymer patches can treat MI through chemical means or by physically stimulating heart regeneration under specific stimuli. For instance, Fan et al. developed a NIR-triggered microneedle patch for MI therapy, which can be easily inserted into the chest cavity through a small incision [191]. Upon NIR irradiation, the patch unfolds and tightly wraps around the heart, delivering VEGF to promote neovascularization. Deng et al. introduced a flexible photonic patch, termed ICarP, for *in vivo* phototherapy [192]. Loaded with chlorella, the patch generates oxygen under specific illumination, addressing hypoxia-related conditions such as MI (Fig. 12A). The local oxygen supply supported by the patch results in healthier myocardium and significantly reduced fibrosis. The myocardium, supported by the local oxygen supply, became healthier. The treated cardiac presented the lowest fibrosis level (20.1 ± 2.5 %) (Fig. 12B), supporting the effects of iCarP triggered *in situ* photosynthesis on fibrosis in reducing fibrosis. Thermal therapy has also shown potential in MI treatment [193]. Feng et al. designed a hydrogel patch that combines thermal therapy with pharmacotherapy [194]. The hydrogel, loaded with PHCuS NPs, generates heat under 808 nm irradiation, promoting controlled copper-ion release and mild photothermal effects (Fig. 12C). This synergistic approach significantly improves cardiac function, as evidenced by the recovery of left ventricular ejection fraction (from 44.9 ± 1.4 % to 72.3 ± 0.7 %) and ventricular fractional shortening (from 20.8 ± 1.1 % to 39.5 ± 1.4 %) (Fig. 12D).

Polymer patches also serve as restoration materials for heart repair. Malki developed nanocomposite scaffolds composed of albumin electrospun fibers and AuNRs, which can convert NIR laser energy to locally alter the fibrous scaffold's molecular structure, securely attaching it to the heart wall [195]. This method minimizes the risk of additional injury associated with conventional stitching techniques. Moreover, Gu designed a MACP as a non-blood-contacting ventricular assist device (Fig. 12E) [196]. Combined with an external MAS, this patch allows for mechanical compression of the heart to assist cardiac circulation (Fig. 12F), offering an alternative solution for myocardial training and therapy in heart failure patients.

While polymer patches present significant advantages in heart therapy, challenges remain. Most patches are invasive, requiring surgery or injection to achieve therapeutic effects. Non-invasive treatment methods for heart diseases warrant further exploration, and the long-term impact of these patches *in vivo* needs continued observation to ensure safety.

### 5.3 Diabetes therapy

Diabetes is a chronic metabolic disease characterized by either insufficient insulin production or impaired insulin uptake. It has emerged as one of the most serious global health concerns, significantly impacting individuals' health and quality of life [197,198]. Traditionally, type 1 and type 2 diabetes are managed by controlling blood glucose levels through insulin pumps or injections of insulin or GLP-1 [199,200]. However, these methods are often painful, inconvenient, and may fail to provide adequate glucose control, increasing the risk of infection. To address these limitations, polymer patches with excellent biocompatibility, particularly polymeric MNs, have been developed for diabetes treatment. Many of these patches are designed to respond to chemical signals such as glucose [201], oxygen [202], H₂O₂ [202,203] and pH [204]. Nevertheless, the complex physiological environments and multi-step chemical responses can lead to unpredictable insulin leakage or delayed blood glucose regulation [205]. Consequently, physical stimulus-responsive patches have garnered significant attention due to their advantages in precise and remote control.

**Fig. 13:**
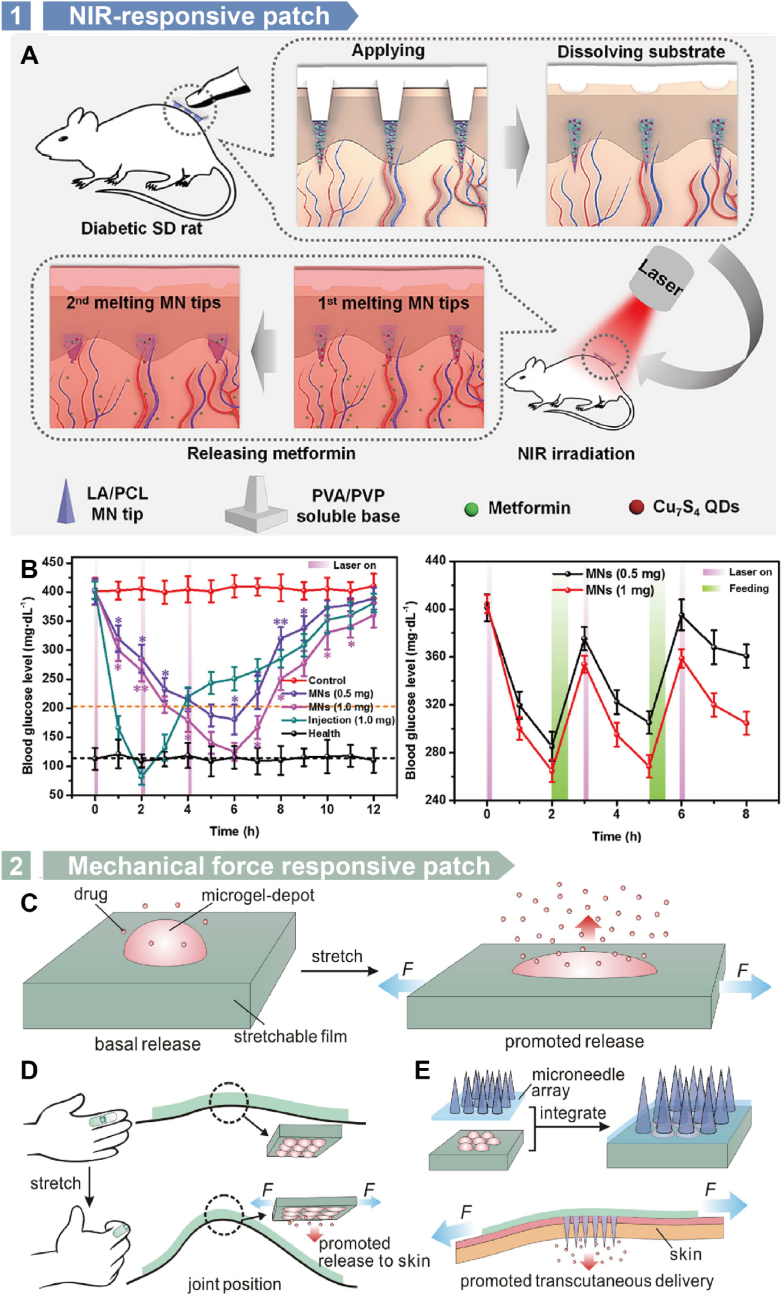
Polymer patches used for diabetes therapy. A) Polymer patch used for NIR-triggered transdermal drug delivery on diabetic SD rats. B) Blood glucose levels of diabetic groups with MN-loaded metformin of 0.5 and 1.0 mg applied under NIR irradiation cycles compared with a hypodermic-injection group with 1.0 mg of metformin and a healthy group, and blood glucose levels of diabetic rats after feeding and NIR exposure. Reproduced with permission [207]. Copyright 2018, AMER CHEMICAL SOC. C) The stretchable elastomeric microgel-depot used for controllable drug delivery. Applying stretches could facilitate the drug release from it. D) Drug loaded wearable patch used for drug delivery. The process can be controlled by finger flexion. E) The drug-loaded patch integrates with a microneedle array patch, which enhances the transcutaneous delivery. Reproduced with permission [49]. Copyright 2015, AMER CHEMICAL SOC.

Among the various stimuli, light-responsive, particularly NIR, patches are favored for diabetes treatment due to their low ionizing potential and high biocompatibility [103,104]. Zhang et al. developed an NIR-triggered polymeric MN patch for diabetes therapy [206]. In this patch, SiO₂ nanoparticles, serving as drug carriers, were coated with PDA and LA to create NIR-responsive drug nanocarriers, which were then encapsulated in PVP MNs. Upon NIR irradiation, LA melts due to the photothermal conversion of PDA, enabling the controlled release of metformin. In vivo studies on diabetic rats confirmed the patch's therapeutic efficacy. In a subsequent design by the same group, the MN patch was made separable [207]. This patch comprised LA/PCL arrowheads and PVA/PVP supporting bases. The drug (metformin) and photothermal conversion factor (Cu₇S₄ nanoparticles) were encapsulated in the LA/PCL arrowheads, which could embed into the skin upon insertion. Under NIR irradiation, the arrowheads melt, facilitating drug release (Fig. 13A). This method allows precise control over the timing and dosage of drug delivery, with the patch maintaining blood glucose levels below 200 mg/dL for over 4.5 h (Fig. 13B), achieving a drug bioavailability of 92.8 % relative to subcutaneous injection.

In addition to light-responsive devices, mechanical force-responsive patches have also been explored for diabetes treatment. Di et al. developed a stretch-triggered drug delivery MN patch composed of a stretchable elastomer and microgel depots containing drug-loaded nanoparticles (Fig. 13C) [49]. When tensile strain is applied to the elastomer film, the drug release is enhanced due to the increased surface area for diffusion and Poisson's ratio-induced compression (Fig. 13D and **E)**. This patch was tested for insulin delivery in diabetic mice, demonstrating that after ten cycles of stretching (with a 50 % strain level), blood glucose levels rapidly dropped to normoglycemic levels (<200 mg/dL) within 30 min, showcasing the potential for on-demand drug delivery. This system could also be adapted for delivering other drugs for various diseases.

While physically responsive polymer patches hold promise for diabetes treatment, several challenges must be addressed to enhance their clinical application. For instance, in NIR-responsive patches, the heat generated may cause the denaturation of macromolecular drugs. Furthermore, other physical stimuli, such as magnetic fields and ultrasound, warrant investigation for controlled drug delivery. Additionally, integrating physically responsive patches with sensors could enable more precise diabetes management [208].

### 5.4 Wound healing

Wound management is a significant global challenge, costing billions of dollars annually [209,210]. The process of wound healing is complex, involving three overlapping but distinct stages: inflammation, new tissue formation, and remodeling [211,212]. During the inflammatory stage, the wound is protected from bacterial infection; however, prolonged inflammation can hinder the healing process. Thus, preventing extended inflammation and promoting angiogenesis are considered critical for effective wound healing [212].

**Fig. 14:**
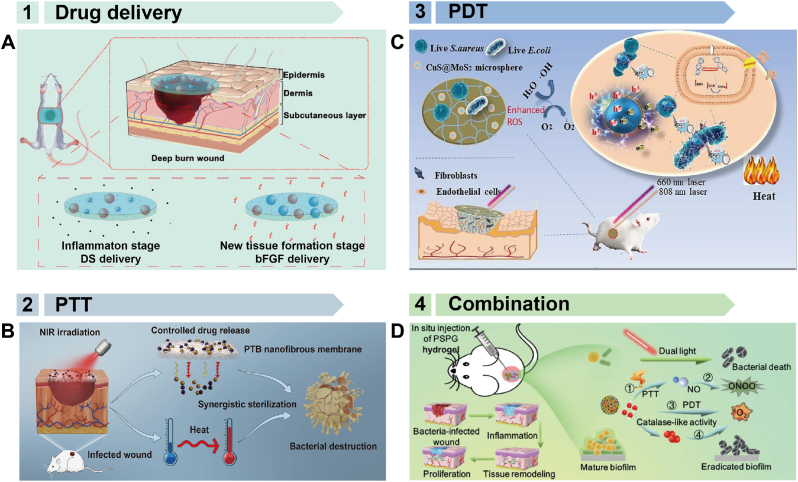
Polymer patches used for wound healing. A) Hydrogel patch utilized to deliver DS and bFGF to suppress inflammation and promote tissue regeneration. Reproduced with permission [214]. Copyright 2020, ELSEVIER. B) Polymer membrane used for wound healing by PTT method. TA-Fe nanoparticles transfer the light to heat to destroy the bacterial cell membrane. Reproduced with permission [217]. Copyright 2019, WILEY. C) Hybrid hydrogel embedded with CuS@MoS_2_ microspheres for wound healing by generating ROS to destroy the bacterial membrane and protein. Reproduced with permission [218]. Copyright 2023, AMER CHEMICAL SOC. D) Injectable hydrogel used for wound healing by combining PTT and PDT. The combination of different therapy strategies enhanced the treatment effect. Reproduced with permission [219]. Copyright 2020, ELSEVIER SCIENCE SA.

One strategy to enhance wound healing is the delivery of drugs such as antimicrobials, antibiotics, anti-inflammatory agents, or growth factors. Recently, various exogenous stimulus-responsive polymeric devices have been utilized for this purpose. For example, the Montaser group designed a thermosensitive hydrogel for controlled drug release in re-infected wounds [213]. This hydrogel was formulated by blending PVA with SA-g-NIPAM, allowing the *in vitro* release of DS to be controlled by external temperature and pH. Similarly, the Lin group developed a thermosensitive hydrogel for wound repair [214]. This hydrogel enables the stepwise delivery of DS and bFGF during the inflammation and new tissue formation stages, respectively (Fig. 14A). *In vivo* studies on rats demonstrated that this composite hydrogel significantly improved wound healing, achieving a 96 % wound contraction at 14 days, with reduced inflammation and enhanced angiogenesis. The entire process could be controlled by the different temperature characteristics of the inflammation and new tissue formation stages.

Another approach to wound healing involves using physical methods to eliminate bacteria, such as PTT and PDT. PTT utilizes the photothermal effect of a photothermal agent to generate heat and destroy abnormal cells, while PDT employs light-responsive photodynamic agents with specific excitation sources to generate ROS for killing microorganisms [215,216]. Both are minimally invasive techniques that can be applied using exogenous stimulus-responsive polymeric devices. For instance, Lin and colleagues developed a NIR light-responsive bifunctional botanical nanofibrous membrane for wound healing [217]. This membrane comprised PVA as a support matrix, tannic TA-Fe (III) nanoparticles as photothermal agents, and BBH as an antibacterial drug. Under NIR irradiation, the TA-Fe nanoparticles rapidly convert light energy into heat, which destroys bacterial cell membranes (Fig. 14B). The heat also accelerates the release of BBH from the nanofibrous membrane, resulting in a synergistic antibacterial effect and promoting wound healing. *In vivo* studies in mice treated with the patch demonstrated a healing rate of 89.02 ± 3.29 % and an antibacterial rate of 94.35 ± 2.01 %. In another study, Zhang et al. synthesized a hybrid hydrogel embedded with CuS@MoS₂ microspheres for wound healing [218]. When exposed to NIR, the hydrogel generates ROS to destroy bacterial membranes and proteins (Fig. 14C). Additionally, under dual light (660 nm + 808 nm) irradiation, the hydrogel produces heat to further kill bacteria. This synergistic PTT and PDT approach achieved a 99.3 % reduction in *E. coli* and a 99.5 % reduction in *Staphylococcus aureus* within 15 min, showcasing the potential for treating bacteria-infected wounds.

Currently, combining different therapeutic modalities in a single device has become a significant research focus. For example, the Du group developed an injectable hydrogel for bacterial biofilm eradication and wound healing [219]. The hydrogel's nanoplatform was constructed by loading photothermally sensitive SNP into a platinum-modified porphyrin metal-organic framework (PCN) and *in situ* modification with gold particles. This platform facilitated the continuous decomposition of endogenous hydrogen peroxide (H₂O₂) into oxygen, enhancing the PDT effect. Additionally, under dual NIR irradiation, the PSPG hydrogel generated hyperthermia (η = 89.21 %), produced reactive oxygen species, and triggered nitric oxide (NO) release, contributing to biofilm destruction (Fig. 14D). This combination of therapies reduces the need for antibiotics and offers a promising strategy against antimicrobial resistance and biofilm-associated infections.

Despite the progress in using polymer patches for wound healing, several challenges remain. Most pathological changes in the wound microenvironment occur in deeper tissues, necessitating further research into deeper detection and treatment using patches. The size of the patches also limits the amount of drug that can be delivered, highlighting the need for materials with comprehensive properties and appropriate structures. Furthermore, the development of specific sensors to monitor various indicators of the wound microenvironment is crucial for creating more intelligent and versatile patches.

### 5.5 Cancer treatment

Cancer is one of the most severe diseases threatening human health due to its rapid tumor growth, sudden onset, and systemic spread and metastasis [9,220]. Conventional therapies, including surgical resection, pharmacological chemotherapy, and radiotherapy, have been employed for cancer treatment. However, these methods often require long-term treatment and are associated with serious side effects [221,222]. Therefore, controllable and precise treatment using simple polymer patches has garnered significant attention.

**Fig. 15:**
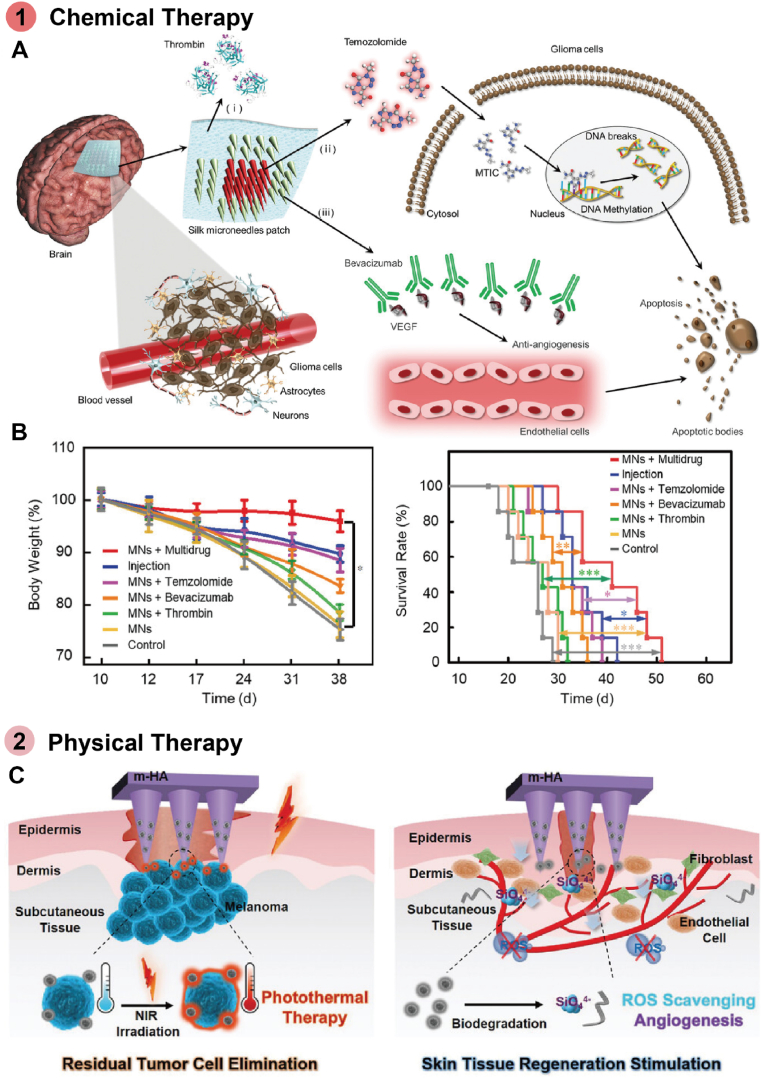
Polymer patches utilized in cancer healing. A) Silk MN patch used to deliver thrombin, temozolomide, and bevacizumab. The drugs are spatiotemporally and sequentially delivered for hemostasis, anti-angiogenesis, and apoptosis of tumor cells. B) Weight change of tumor bearing mice after MNs implantion and Survival analysis of mice determined by the log-rank (Mantel-Cox) test. Reproduced with permission [226]. Copyright 2022, WILEY-V C H VERLAG GMBH. C) MN patch used for skin tumor photothermal therapy and wound healing. The patch generates heat under NIR irradiation to eradicate infiltrating tumor cells by PTT. Besides, the patch could suppress oxidative stress and inflammatory responses of macrophages to promote the recovery of wounds. Reproduced with permission [229]. Copyright 2022, WILEY-V C H VERLAG GMBH.

One approach to cancer treatment is the sustained delivery of drugs. For example, Saeednia group developed a thermosensitive chitosan–graphene hybrid hydrogel for sustained and controllable drug delivery *in vivo* [223]. The inclusion of graphene enhanced the swelling behavior of the hydrogel and reduced the burst release of MTX. Additionally, hydrogels loaded with MTX effectively inhibited the growth of MCF-7 breast cancer cells. Li et al. synthesized a heparin-poloxamer hydrogel by combining LMWH and carboxylated P407 to load DOX (DOX@LR-HP) [224]. This hydrogel exhibited an extended and controlled release profile, with the synergistic action of heparin and DOX showing optimal antitumor efficacy. Furthermore, the combination of multiple chemotherapeutic drugs is considered a feasible approach for reducing side effects [225]. For instance, Wang group developed a SMN patch loaded with two therapeutic drugs (thrombin and temozolomide) and a targeted agent (bevacizumab) (Fig. 15A) [226]. The drug delivery could be remotely triggered by NIR irradiation at specific stages post-implantation. The degradation of the microneedles allowed sustained drug delivery for brain tumor treatment. In terms of therapeutic outcomes, the median survival time of mice treated with the SMN patch loaded with multiple drugs was extended to 41 days, representing an increase of approximately 58 % and 24 %, or about 15 and 8 days, over the control and drug injection groups, respectively (Fig. 15B). These MNs hold potential for clinical applications in the treatment of brain tumors using combined drug therapies.

In addition to drug delivery, physical therapy methods such as PTT and PDT are promising strategies for cancer treatment. Fu group designed a hydrogel containing PB nanospheres, which exhibited excellent serum stability, photothermal conversion efficiency, and stability under repeated laser exposure [227]. This hydrogel demonstrated promising photothermal therapeutic effects with fewer side effects compared to other photothermal agents containing heavy metal elements. They later fabricated a MN patch incorporating the photosensitizer IR820 and the chemotherapeutic agent CDDP for treating breast cancer [228]. IR820 generates singlet oxygen, superoxide anions, and other reactive oxygen species (ROS) upon laser irradiation. The combination of PDT and chemotherapy resulted in better therapeutic outcomes with low cytotoxicity and good biocompatibility. The highest tumor inhibition rate observed *in vivo* was 90.0 %. Lei et al. developed a microneedle patch for simultaneous skin tumor photothermal therapy and wound healing [229]. Natural melanin nanoparticles derived from CINP were loaded into the patch, which possesses both antioxidative and photothermal properties. Under NIR irradiation, the patch facilitated PTT for cancer treatment and scavenged ROS for skin tissue regeneration (Fig. 15C). After treatment with the MNs patch and 808 nm laser, most tumors either diminished in size or disappeared without recurrence by day 9, and the original surgical wound nearly closed with a small scab by day 12. This patch holds significant potential as a supplementary therapy following skin tumor resection. Other physical methods, such as MHT [230] and ultraviolet therapy [231], also offer potential strategies for cancer treatment.

Despite the significant progress in developing patches for cancer treatment, several challenges remain. Many types of cancer occur in deeper tissues, necessitating the design of non-invasive patches for deeper treatment to minimize their impact on the body. For more precise therapy, these patches could be combined with targeting drugs to improve therapeutic efficacy and reduce side effects. Additionally, biological barriers, such as the blood-brain barrier, may obstruct cancer treatment. To address this, patches could incorporate delivery strategies that facilitate the transport of therapeutics across these barriers [232]. The application of nanocarriers offers a potential solution, as polymer patches could be used to load stimulus-responsive nanocarriers, which can be combined with specific drugs for cancer treatment [233]. Due to the passive and active targeting capabilities of nanocarriers, therapeutic efficacy could be enhanced, and side effects minimized [234]. Furthermore, surface functionalization of nanocarriers with targeting ligands can enable receptor-mediated transcytosis across the blood-brain barrier, increasing the local concentration of drugs [235]. The combination of polymer patches and nanocarriers holds promise for improving cancer treatment.

### 5.6 Obesity treatment

Obesity, characterized by excessive fat accumulation in adipose tissue, has become a significant public health issue. Beyond the inconvenience it causes in daily life, obesity is linked to various diseases, including diabetes, cardiovascular diseases, and cancer [[236], [237], [238]]. Current treatment options primarily include surgeries and oral medications [239,240]. However, drug therapies require strict adherence, and surgeries may have detrimental consequences on the body [241]. Therefore, a simple and effective treatment strategy for obesity is needed, and polymer patches present a promising alternative due to their safety and simplicity.

WAT and BAT are the two main types of adipose tissue in mammals [242]. WAT stores excess metabolic energy as triglycerides, while BAT utilizes this energy from metabolically active brown adipocytes to generate heat, thereby reducing body fat accumulation [243]. A potential treatment strategy involves the browning of white fat, which transforms WAT into BAT.

**Fig. 16:**
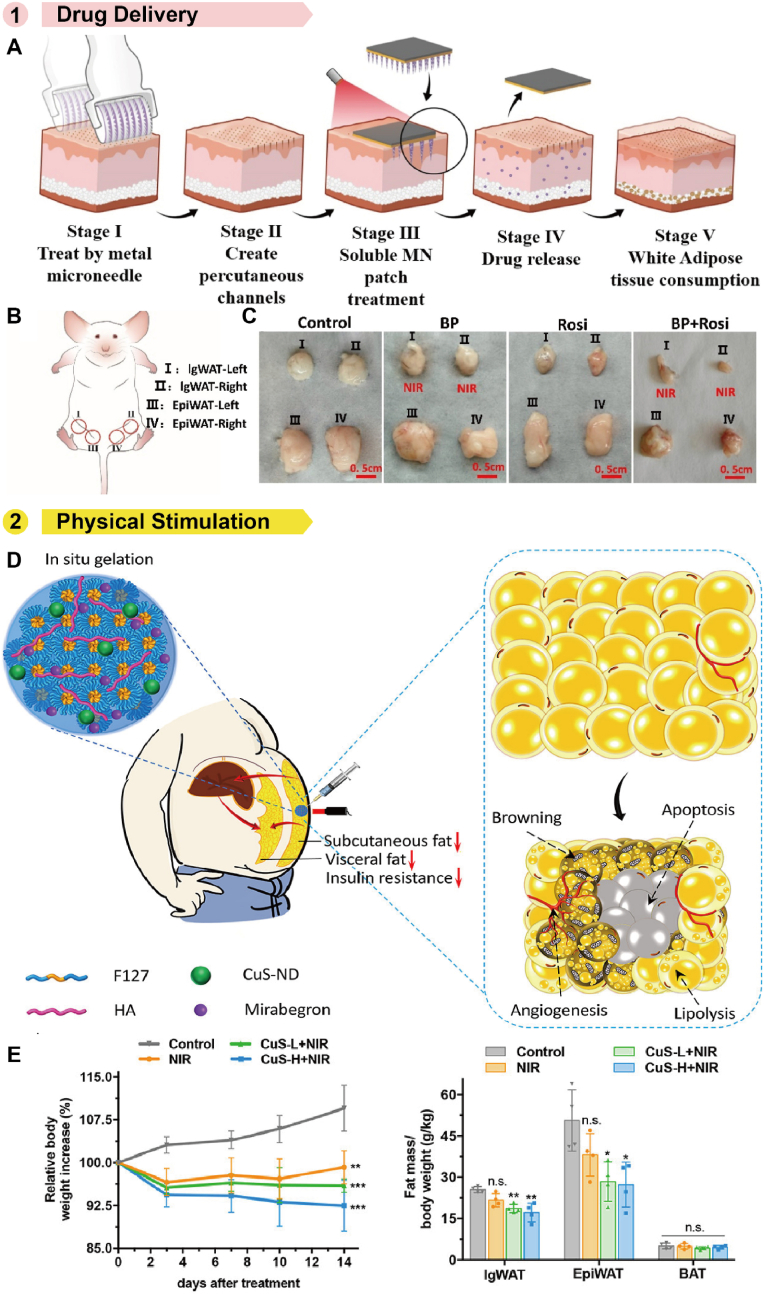
Polymer patches used for obesity treatment. A) Treatment process of NIR-responsive MN patch, which could deliver Rosi for body slimming. B) Adipose tissue of C57 mice. I: Left side of inguen white adipose tissue (IgWAT-Left); II: Right side of inguen white adipose tissue (IgWAT-Right); III: Left side of epididymis white adipose tissue (EpiWAT-Left); IV: Right side of epididymis white adipose tissue (EpiWAT-Right). C) Images of adipose tissues collected after dissection of mice. Reproduced with permission [245]. Copyright 2020, ELSEVIER. D) Hydrogel utilized for transdermal photothermal-pharmacotherapy of obesity. E) Relative body weight changes over the course of treatment and relative fat mass at day 14. Reproduced with permission [248]. Copyright 2022, AMER CHEMICAL SOC.

Some polymer patches have the capability to deliver drugs, such as Rosi, to promote the browning of WAT. For example, the Peng group developed a near-infrared (NIR)-responsive soluble MN patch to deliver Rosi for body slimming [244]. Rosi acts as a weight-loss drug, while melanin is added to enhance the photothermal effect and accelerate drug release to the targeted fat region under NIR light. When combined with external cold stimulation to boost fat consumption, the patch achieved significant and precise body slimming effects. They also developed a black phosphorus-modified soluble MN patch to deliver Rosi (Fig. 16A) [245]. The black phosphorus layer exhibited excellent photothermal properties and biocompatibility. Under NIR irradiation, the release of Rosi was accelerated, promoting the lysis of fat cells for obesity treatment (Fig. 16C). After treated with the patch and an 808 nm laser, the weights, volumes of adipose tissues, and average waist circumference of mice decreased, demonstrating the patch's effectiveness in achieving targeted body slimming.

In addition to chemical drugs, mild heating of adipose tissue can effectively stimulate the transition of WAT to BAT [246]. Some polymer patches utilize PTT to treat obesity. Gao group designed a hyaluronic acid MN patch loaded with PDA-NPs and mirabegron for weight loss [247]. PDA-NPs, as an excellent photothermal conversion agent, converted light into heat energy when exposed to NIR, inducing the browning of WAT. The weight of obese mice decreased by nearly 19 % after photothermal treatment. When combined with the chemical drug mirabegron, weight loss was more significant, with a reduction of about 22 %, demonstrating the patch's potential for body slimming. Similarly, Zan group developed an injectable hydrogel to remodel adipose tissue [248]. Loaded with the photothermal conversion agent CuS-NDs, the hydrogel generated heat to induce the browning of WAT (Fig. 16D). When combined with mirabegron, the hydrogel exhibited strong therapeutic synergy, completely inhibiting obesity development in high-fat diet-fed mice, resulting in a 15 % reduction in body weight and decreased subcutaneous (40 %) and visceral fat masses (54 %) (Fig. 16E). In addition to PTT, MHT also shows potential in treating obesity. Su et al. developed a Fe_3_O_4_ microsphere-doped hydrogel for obesity therapy, which generated heat under specific magnetic fields to promote lipolysis in white adipocytes. These physical methods offer promising alternatives to traditional treatments for obesity.

However, the use of thermotherapy may affect other tissues, raising concerns about potential risks to surrounding tissues. Further research is needed to address these risks. Additionally, these methods primarily target adipose tissue in localized areas, such as abdominal fat, and the effects of therapy on whole-body metabolism require further investigation.

### 5.7 Hair growth

Hair loss has become a prevalent disorder in humans, attributable to various factors such as aging, disease, and medications [249]. Current therapies primarily involve hair follicle transplantation, a method significantly constrained by the high cost, invasive nature of the surgery, and a limited supply of donors [250]. A promising alternative involves the use of exosomes or Mx to stimulate intrinsic hair follicle regrowth. However, this approach necessitates the sustained delivery of these therapeutic agents [251,252]. As a result, polymer patches designed for sustained release have garnered significant attention.

**Fig. 17:**
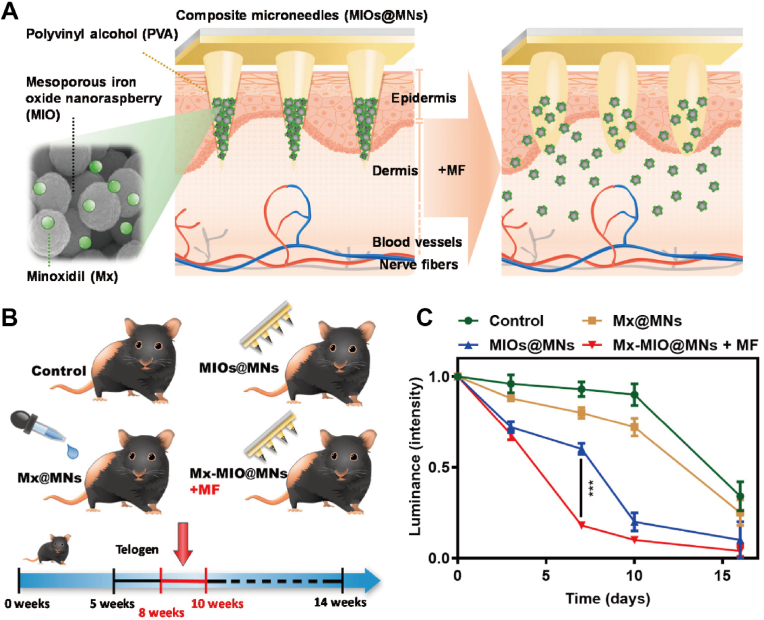
Polymer patches used for hair growth. A) MN patch delivering Mx triggered by an external magnetic field. B) Schematic illustration represents *in vivo* evaluation of MN patch for hair loss treatment. C) The hair intensity treated by control, Mx@MNs, Mx-MIOs@MNs, and Mx-MIOs@MNs + MF groups for hair growth on mice. Reproduced with permission [253]. Copyright 2020, MDPI.

Several patches have been developed for the sustained delivery of Mx. For instance, Fang et al. designed a MN patch using PVA for the treatment of androgenetic alopecia [253]. The patch incorporates magnetic mesoporous nanoparticles composed of silica and iron oxide (MIO), which generate heat under an external magnetic field to control the release of Mx (Fig. 17A). In addition to controlling drug delivery, the heat induces increased cutaneous blood flow and vasodilation, thereby promoting hair growth. Sustained delivery of exosomes has also been employed to enhance hair growth. In a study, mice treated with MN patches showed an 800 % increase in hair density 10 days post-treatment compared to untreated mice (Fig. 17C). Xiong group utilized ADSC-NVs to deliver JAM-A protein for hair regeneration [254]. The ADSC-NVs and overexpressed JAM-A were encapsulated in a thermosensitive hydrogel patch, creating a sustained-release platform (JAM-A^OE^@NV Gel). This platform activates the autophagy and Wnt/β-catenin pathways in DPCs in response to DHT and macrophage-induced damage. This innovative approach not only offers a novel perspective on cell-free regenerative therapies for AGA but also holds significant potential for clinical application and translation.

Although numerous patches have been developed for hair growth, many still rely on the inherent properties of materials rather than physical control mechanisms to achieve sustained drug release, potentially limiting the range of materials that can be utilized. Additionally, combining physical therapy methods, such as phototherapy, with polymer patches may enhance therapeutic outcomes.

### 5.8 Scar therapy

HS results from an abnormal healing response to trauma, inflammation, or burns, characterized by persistent dermal fibrosis, irregular collagen deposition, and excessive proliferation of HSF [255,256]. HS not only affects the patient's appearance but also causes symptoms such as redness, thickening, pain, and itching in the affected tissue [257]. Conventional treatment methods, including surgical resection, laser excision, and topical silicone sheeting, face significant challenges, such as low efficacy, high recurrence rates, and painful procedures. Consequently, there is growing interest in novel therapies based on polymer patches.

**Fig. 18:**
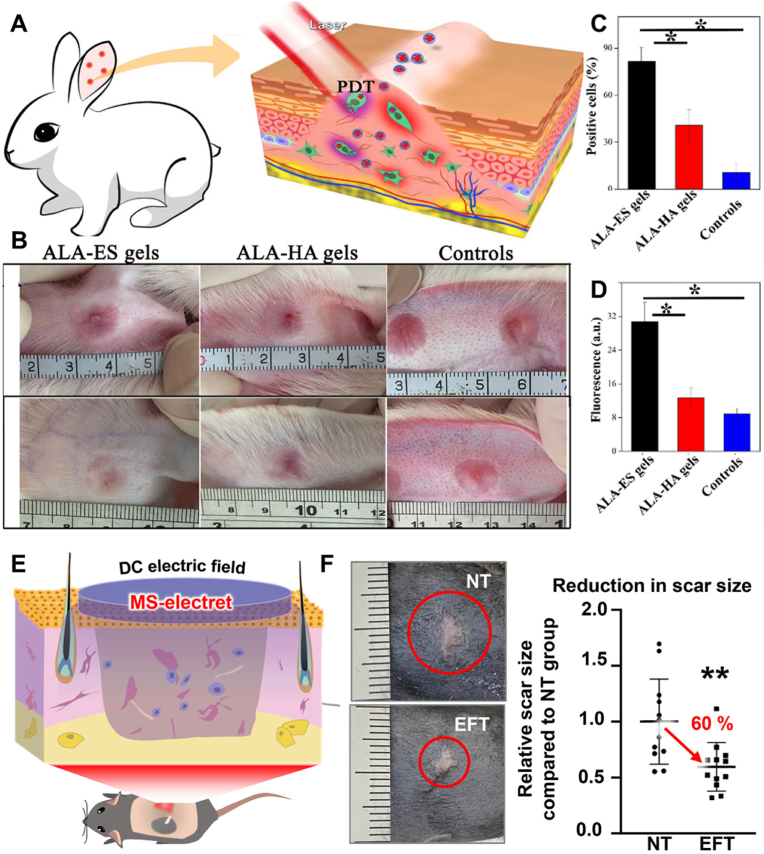
Polymer patches utilized for scar therapy. A) Application of ALA-ES gels for *in vivo* PDT. B) Appearance changes of HS in different groups before and after four PDT sessions. C) TUNEL staining positive HSF cells in the dermis. D) Statistics of mean MMP-3 immuno-fluorescence of HSF cells. Reproduced with permission [258]. Copyright 2019, AMER CHEMICAL SOC. E) Illustration of multilayer stacked-electret patch for inhibition of scar formation. F) Appearance changes of HS and scar size in different groups after applying electrical stimulation or not. Reproduced with permission [259]. Copyright 2024, WILEY.

One promising therapeutic approach is PDT, which induces apoptosis of HSFs and remodels collagen fibers to treat HS. For instance, Zhang et al. developed a gel loaded with ALA for HS treatment (Fig. 18A) [258]. The ALA was encapsulated in nanoethosomes to create ALA-ES gels, which can penetrate rabbit HS tissue, facilitating the accumulation of ALA in HSFs. Compared with ALA-HA gel, the penetration of ALA-ES after 6 h was approximately 30 % higher. Following the application of a 635 nm laser, the apoptosis rate of HSFs reached 83 ± 9.0 % (Fig. 18C), demonstrating the efficacy of PDT in promoting HSF apoptosis and remodeling collagen fibers using the ALA-ES gel.

In addition to PDT, electrical stimulation has also been explored for scar therapy. Gin et al. developed a multilayer stacked electret patch for inhibiting skin scar formation (Fig. 18E) [259]. The patch, composed of multiple layers of FEP dielectric film fabricated by a corona charging system, generates a DC EF without the need for external power. By stacking electret layers, the intensity of the EF can be easily controlled, maintaining a stable EF with a surface potential of 3400 V for over 5 days. As a result, the relative scar size was reduced by nearly 60 % (Fig. 18F). This patch shows great potential for clinical applications due to its ability to create a stable and customizable electrophysiological microenvironment for scar inhibition without requiring external power.

Although these patches have shown significant potential for scar therapy, several challenges remain. The long-term effects of stimulation require further investigation to ensure therapeutic efficacy and minimize side effects. Moreover, the mechanisms underlying some methods, such as electrical stimulation, remain largely unexplored. Further research is needed to fully understand the potential of EFs in scar and wound healing.

### 5.9 Other diseases

**Fig. 19:**
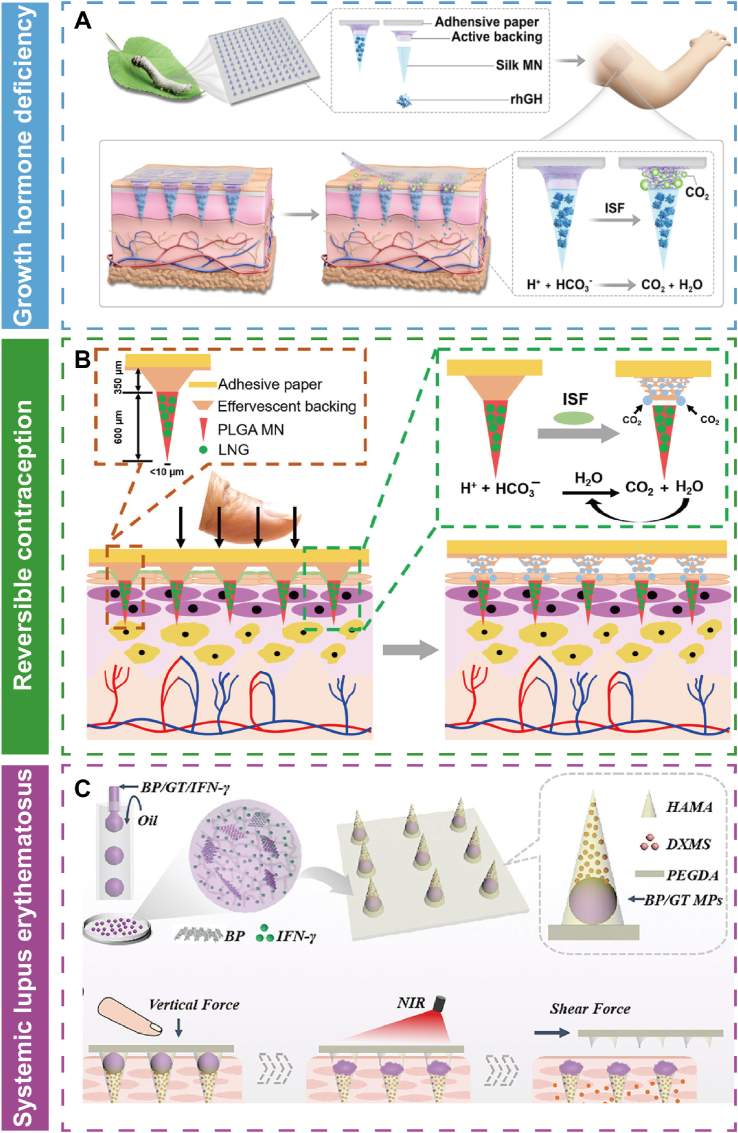
Treatment of other diseases by using polymer patches. A) Mechanical force-responsive silk MN patch used to deliver growth deficiency. Reproduced with permission [260]. Copyright 2023, INST MATERIA MEDICA, CHINESE ACAD MEDICAL SCIENCES. B) Separable MN patch used for long-term reversible contraception. Reproduced with permission [261]. Copyright 2019, AMER ASSOC ADVANCEMENT SCIENCE. C) NIR-trigger MN patch utilized for systemic lupus erythematosus therapy. Reproduced with permission [262]. Copyright 2022, Photothermal Responsive Microspheres-Triggered Separable Microneedles for Versatile Drug Delivery.

Polymer patches have also been employed to treat various diseases. Yang et al. developed a mechanically triggered MN patch for treating growth hormone deficiency [260]. The patch, composed of silk protein, allows for the sustained release of rhGH. After manual application of pressure for 1 min, the needles detach from the backing paper by absorbing interstitial fluid, which activates bicarbonate ions (HCO^3−^) in the active backing layer to produce carbon dioxide gas (Fig. 19A). The detached MNs then maintain the sustained release of rhGH for more than 7 days, producing effects comparable to daily injections. Utilizing a similar mechanism, Li et al. designed a separable MN patch for long-acting reversible contraception [261]. Upon applying pressure for 1 min, sodium bicarbonate and citric acid in the patch's backing layer react with interstitial fluid in the skin, generating carbon dioxide bubbles. These bubbles weaken the attachment of the MNs to the patch, enabling their separation (Fig. 19B). The polymer patch demonstrated the capability to release levonorgestrel for 1 month both *in vitro* and *in vivo* in rats, indicating significant potential as a long-acting contraceptive. Additionally, polymer patches have been explored for treating systemic lupus erythematosus. Fan et al. developed a NIR-triggered separable MN patch to deliver interferon γ and dexamethasone [262]. The patch integrates photothermally responsive phase-changing MPs composed of BP and phase-changing GT into conical hydrogel MNs. Upon NIR irradiation, the MPs generate heat, forming cavities in the MNs that allow for easy detachment of the backing plate from the needles via shear force (Fig. 19C). This controllably separable MN patch effectively delivers two drugs for systemic lupus erythematosus therapy. After treatment with the MN patch, results showed a progressive reduction in IL-6 and creatinine levels, indicating a decrease in inflammatory cells.

Based on the studies discussed, we have summarized the biomedical applications of physical stimulus-responsive polymer patches in Table 3. These patches have shown responsiveness to physical stimuli and therapeutic efficacy across various diseases. However, several issues related to research methods warrant discussion. First, the majority of studies were conducted using animal models, particularly mice. Due to the physiological differences between mice and humans, some evaluation metrics, such as blood drug concentration, may not provide sufficient relevance for human treatment. Additionally, most studies assessed biocompatibility solely through cytotoxicity tests, with limited consideration of other critical factors such as hemocompatibility and genotoxicity. Furthermore, the long-term effects of these patches require thorough investigation. Lastly, to ensure the responsiveness of these patches, real-time monitoring of indicators such as drug concentration and blood glucose levels is essential. Real-time monitoring, as opposed to intermittent assessment, can yield more valuable insights into the treatment process, particularly regarding the sensitivity of patches to specific physical stimuli.

## 6 Challenges

**Fig. 20:**
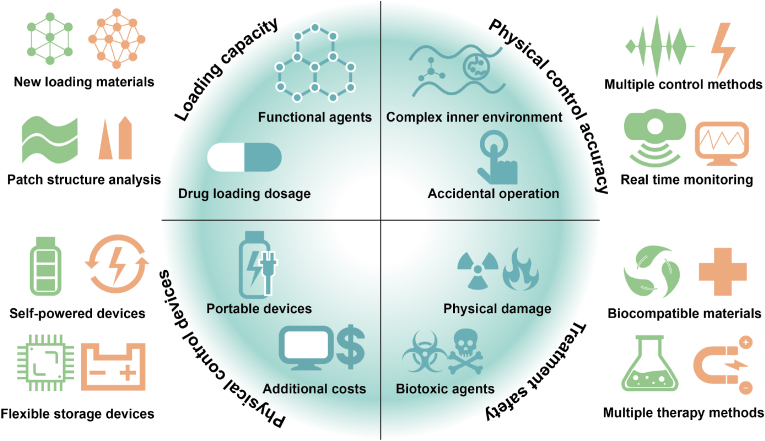
Challenges and prospects of physical stimulus-responsive polymer patches. The main challenges of polymer patches include loading capacity, physical control accuracy, additional control devices, and treatment safety. The potential solutions include materials design and analysis, combination of functions, combined control methods, and multiple therapy strategies.

Although physical stimulus-responsive polymer patches show significant potential for healthcare applications, several challenges still hinder their broader implementation. We have summarized the primary challenges, as illustrated in Fig. 20.

### 6.1 Loading capacity

While many polymer patches have demonstrated significant therapeutic effects, their treatment duration typically lasts only a few days or even hours, necessitating additional drug dosages for extended treatment. Furthermore, most studies have been conducted on experimental animals, whose physiological conditions often differ from those of humans, leaving the actual therapeutic period untested. Additionally, increasing the loading capacity of patches to accommodate more functional components remains a significant challenge, particularly when higher doses are required.

### 6.2 Accuracy of physical control

The effectiveness of therapy is largely determined by the control of treatment. However, due to the varying and complex internal environments of different patients, treatments governed by identical physical conditions can produce different outcomes. Enhancing the sensitivity and adaptability of patches across diverse patient profiles remains a significant challenge. Additionally, certain control methods, such as those relying on mechanical force, depend on patient operation. Incorrect use or accidental activation may reduce therapeutic efficacy and increase the risk of medical complications.

### 6.3 Physical control devices

Many physical control methods depend on external devices such as light sources, magnets, and batteries, which can impose additional burdens on patients, potentially making these treatments unaffordable. Furthermore, the design and fabrication of portable external stimuli sources present significant challenges that need to be addressed.

### 6.4 Treatment safety

While many polymer patches effectively treat diseases through drug delivery or physical methods, there are still significant risks associated with their use, such as drug toxicity and physical damage (e.g., light radiation burns), which can compromise the safety of the therapy. Additionally, many functional polymer patches incorporate various nanomaterials, but the long-term biodegradability and clearance of these materials, particularly in invasive patches, remain concerns. Further investigation is required to enhance the safety of these therapies.

## 7 Prospects

To solve these problems, several potential solutions have been given in this section (Fig. 20). To increase the loading capacity of patches, not only new materials should be explored, but also attention should also be given to the structural design of the patches. Studies have shown that adopting a finite element approach, which utilizes mathematical models to simulate real physical systems, including geometry and loading conditions, may help address loading issues. Furthermore, combining different therapeutic methods, particularly chemotherapy with physical methods such as PTT and PDT, could enhance therapeutic efficacy and reduce the required drug dosage. However, the stability of drugs and functional components in varying physical and chemical environments may pose a challenge to implementation.

For improving the precision of physical control, a potential strategy is the development of multi-stimuli-responsive systems. Such systems could integrate multiple stimuli, such as light and electric fields, as inputs, thereby reducing reliance on a single stimulus and increasing the sensitivity of patches. Additionally, patches could be equipped with biosensors to monitor relevant physiological indicators, such as glucose levels, sweat composition, and temperature, further enhancing control. However, these methods typically require additional devices, particularly for sourcing different physical stimuli. The miniaturization and integration of these devices remain significant challenges.

Regarding additional devices, self-powered systems may be a viable option for the long-term use of patches. Power could be generated by loading components or converting other forms of energy, such as PENG and TENG. To extend the usage period of patches, flexible energy storage devices could also be employed. However, current nanogenerators are unable to consistently provide stable electrical energy, which may compromise the therapeutic effect and remains a challenge to be addressed.

Finally, in terms of treatment safety, developing of new functional nanomaterials with high biocompatibility is essential. Combining multiple therapeutic methods to mitigate the side effects of monotherapy is another potential strategy to enhance treatment safety.

In addition to these solutions, emerging technologies may also guide the development of polymer patches. For example, artificial intelligence could be leveraged to predict and design functional materials for physical stimulus-responsive polymer patches. Additionally, 4D printing technology could be used to precisely fabricate polymer patches with complex structures. These innovations will likely drive advancements in the biomedical application of polymer patches.

## 8 Conclusion

**Fig. 21:**
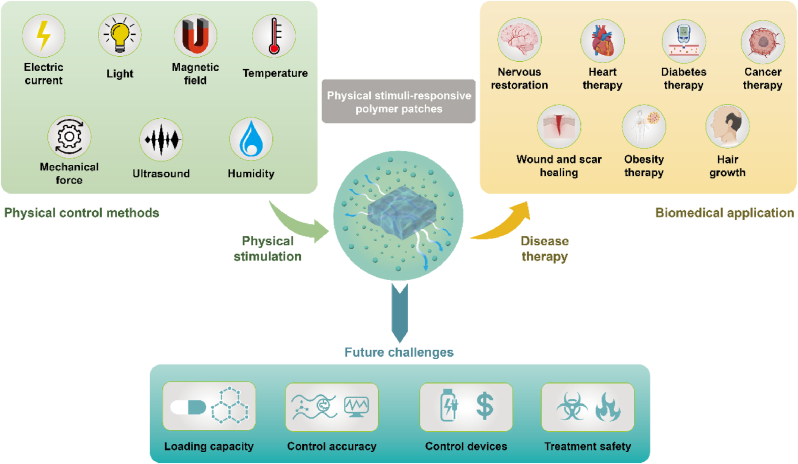
Summary of the recent advances of physical stimuli-responsive polymer patches, including physical control methods, biomedical application and future challenges.

This review summarizes recent advances in physical stimulus-responsive polymer patches, which hold significant potential for healthcare applications due to their biocompatibility, processability, and cost-effectiveness (Fig. 21). The design principles of these patches were outlined to provide useful references, and the mechanisms of various physical control methods were introduced, along with their advantages and limitations. Additionally, the fabrication methods for polymer patches were discussed to guide further research. Recent applications of these patches in the biomedical field were also reviewed, demonstrating that treatment under specific physical stimuli can be effectively controlled through chemotherapy or physical therapy, resulting in a controllable therapeutic process with significant efficacy. Overall, physical stimuli-responsive polymer patches show great promise in biomedical applications.

Despite these advantages, several challenges remain, including loading capacity, control accuracy, device integration, and therapy safety. Loading capacity directly impacts treatment duration and functionality, and patches with higher drug and functional component loads can offer prolonged treatment and enhanced therapeutic functions. Control accuracy is crucial for ensuring the precise management of treatments, reducing the risk of overtreatment, and improving therapeutic outcomes. The integration of control devices is essential for enabling controllable therapy, and the miniaturization and simplification of these devices are vital for maintaining low costs and enhancing user convenience. Therapy safety is paramount for patient health and is closely tied to the biocompatibility of materials and the potential for treatment-related damage. Although existing patches have demonstrated promising results in some areas, these challenges still limit their practical application. To address these issues, further research is needed into the development of functional materials with enhanced biocompatibility and multifunctionality, as well as the structural optimization of polymer patches. Additionally, the design and utilization of miniature devices integrated with patches should be prioritized. The advancement of polymer patches with improved functionalities and biocompatibility has become a focal point of research. Emerging technologies, such as AI and 4D printing, may also play a critical role in the continued development of these patches.

Physical stimulus-responsive polymer patches have made considerable progress in biomedical research, showing immense potential in the areas of personalized treatment and on-demand drug delivery. Despite facing challenges related to clinical translation and commercialization, current studies are paving the way for safe and highly efficient therapeutic methods. With the integration of new technologies, including bioelectronics and AI, these innovative biomedical polymer patches are poised to benefit society in the near future.

## Ethics approval and consent to participate

This manuscript is a literature review work, and thus no *in vivo* evaluations on animal model or clinical trials were performed in this scope. Thereby, our work does not fall into the incidence of ethical approvals and patient consents.

## CRediT authorship contribution statement

**Yifan Cheng:** Writing – review & editing, Writing – original draft, Methodology, Investigation, Formal analysis. **Yuan Lu:** Writing – review & editing, Supervision, Funding acquisition, Conceptualization.

## Declaration of competing interest

The authors declare that they have no known competing financial interests or personal relationships that could have appeared to influence the work reported in this paper.
